# A novel lncRNA MDHDH suppresses glioblastoma multiforme by acting as a scaffold for MDH2 and PSMA1 to regulate NAD+ metabolism and autophagy

**DOI:** 10.1186/s13046-022-02543-7

**Published:** 2022-12-17

**Authors:** Dong He, Tao Xin, Bo Pang, Jun Sun, Zi Hao Liu, Zhen Qin, Xiao Shuai Ji, Fan Yang, Yan Bang Wei, Zi Xiao Wang, Jia Jia Gao, Qi Pang, Qian Liu

**Affiliations:** 1 Department of Neurosurgery, Shandong Provincial Hospital, Shandong University, Jinan, 250012 Shandong P.R. China; 2 Department of Neurosurgery, Shandong Provincial Hospital Affiliated to Shandong First Medical University, Jinan, 250012 Shandong P.R. China; 3 Department of Histology and Embryology, Cheeloo College of Medicine, School of Basic Medical Sciences Shandong University, Jinan, 250012 Shandong P.R. China; 4 Department of Neurosurgery, Shandong Provincial Qianfoshan Hospital, Shandong First Medical University & Shandong Academy of Medical Sciences, Shandong Medicine and Health Key Laboratory of Neurosurgery, Jinan, 250014 P.R. China; 5 Department of Neurosurgery, Shandong Provincial Qianfoshan Hospital, Cheeloo College of Medicine, Shandong University, Jinan, 250014 P.R. China; 6 Department of Clinical Laboratory, Affiliated Hospital of Shandong University of Traditional Chinese Medicine, Jinan, 250012 Shandong P.R. China

**Keywords:** Glioblastoma multiforme, MDHDH, MDH2, Metabolism-based therapy, Molecular scaffolding

## Abstract

**Background:**

To identify potential targets related to nicotinamide adenine dinucleotide (NAD+) metabolism in gliomas, we used RNA immunoprecipitation to identify a novel long noncoding RNA renamed malate dehydrogenase degradation helper (MDHDH) (NONCODE annotation ID: NONHSAT138800.2, NCBI Reference Sequence: NR_028345), which bound to MDH2 (malate dehydrogenase 2), that is downregulated in glioblastoma multiforme (GBM) and associated with metabolic regulation. However, its underlying mechanisms in the progression of GBM have not been well studied.

**Methods:**

To investigate the clinical significance of MDHDH, we analyzed its expression levels in publicly available datasets and collected clinical samples from Shandong Provincial Hospital, affiliated with Shandong University. Functional assays, including FISH/CISH, CCK8, EdU, wound healing, and transwell assays, were used to determine the cellular/subcellular localization, tissue expression profile and anti-oncogenic role of MDHDH. Furthermore, RNA pulldown, mass spectrometry RNA immunoprecipitation, coimmunoprecipitation, JC-1 probe, and cell energy-production assays were used to determine the mechanisms of MDHDH in the development of GBM. Animal experiments were conducted to determine the antitumorigenic role of MDHDH in GBM in vivo.

**Results:**

In public datasets, MDHDH expression was significantly downregulated in GBM and LGG compared with GTEx normal brain tissues. The results of the tissue microarray showed that the MDHDH expression level negatively correlated with the tumor grade. Altered MDHDH expression led to significant changes in the proliferation, migration and invasion of GBM cells both in vitro and in vivo.

Mechanistically, we found that MDHDH directly bound to MDH2 and PSMA1 (20S proteasomal core subunit alpha-type 1) as a molecular scaffold and accelerated the degradation of MDH2 by promoting the binding of ubiquitinated MDH2 to the proteasome. The degradation of MDH2 subsequently led to changes in the mitochondrial membrane potential and NAD+/NADH ratio, which impeded glycolysis in glioma cells.

**Conclusions:**

In conclusion, this study broadened our understanding of the functions of lncRNAs in GBM. We demonstrated that the tumor suppressor MDHDH might act as a clinical biomarker and that the overexpression of MDHDH might be a novel synergistic strategy for enhancing metabolism-based, epigenetic-based, and autophagy regulation-based therapies with clinical benefits for glioblastoma multiforme patients.

**Supplementary Information:**

The online version contains supplementary material available at 10.1186/s13046-022-02543-7.

## Introduction

Glioma is acknowledged as the most prevalent malignant brain tumor and, according to the WHO classification of Central Nervous System (CNS) Tumors published in 2016, glioblastoma multiforme (GBM) is one of the most lethal forms, with an average survival time of only 12-15 months [1–4]. Due to the aggressive and invasive nature of glioma, resecting tumor tissues completely in surgery is considered to be impractical [5, 6]. Additionally, GBM is characterized by rapid progression, a high relapse rate and poor prognosis attributed to easily induced radio/chemotherapeutic resistance [3]. Therefore, understanding the pathogenesis of glioblastoma and exploring effective therapeutic targets are imperative to GBM treatment.

Altered cellular metabolism is a hallmark of gliomas. Propelled by a set of recent technological advances, new insights into the molecular mechanisms underlying glioma metabolism have rapidly emerged [7–14]. Cancer cells have unique dependencies on NAD+ metabolic pathways. NADH (reduced nicotinamide adenine dinucleotide), which is oxidized to NAD+ to maintain a high rate of glycolysis, can pathologically lead to a typical metabolic alteration of cancer cells that is well known as the “Warburg effect”. NAD+ is recognized as an essential cofactor and substrate for a multitude of biological processes. It is also the currency of metabolic transactions that are critical for cell survival. A previous study confirmed that targeting NAD+ can be an effective way to enhance the metabolic lethality of alkylation chemotherapy in IDH (isocitrate dehydrogenase)-mutant tumor cells. The malate-aspartate shuttle (MAS) is involved in the glycolytic process and NAD+ metabolism. As an indispensable regulatory system for NAD+ metabolism, MAS determines whether impermeable cytosolic NADH can be transported by malate carriers into mitochondria, where 20%-80% of NADH is oxidized in several types of cancer cells [15]. The MAS is operated by two pairs of enzymes localized in the mitochondria and cytoplasm: glutamate oxaloacetate transaminases (GOT1/2) and malate dehydrogenases (MDH1/2) [15]. Mitochondrial malate dehydrogenase (mito-MDH), encoded by *MDH2*, plays a pivotal role in the conversion of malate into oxaloacetate and acts as a key hub in the Krebs cycle, anaerobic glycolysis and oxidative phosphorylation. Researchers have demonstrated that MDH2 could be applied as an effective metabolic therapeutic target based on its enzymatic functions in vitro [16–18]. Therefore, MDH2 inhibition provides a valuable platform for developing novel therapeutics that target cancer metabolism and tumor growth. However, studies on the precise mechanisms of MDH2 posttranslational regulation and its potential effects on NAD+ metabolism are still lacking, especially in glioma.

Dysregulated transcripts, including mRNAs, microRNAs (miRNAs), lncRNAs (long noncoding RNAs), and circular RNAs (circRNAs), can be a primary feature in human cancers [19–23]. With the rapid development of high-throughput RNA sequencing and wide application of bioinformatics, lncRNAs, as a subset of ncRNAs with length >200 nucleotides, were identified to participate in diverse biological processes in cancers [19]. LncRNAs can serve as signal mediators, scaffolds or molecular decoys to function in epigenetic, transcriptional and posttranscriptional regulation. Specifically, the interaction between lncRNAs and the ubiquitin (Ub)-proteasome system (UPS) has attracted wide attention from researchers. For instance, lncRNA AGPG protected PFKFB3 by preventing APC/C-mediated ubiquitination from proteasomal degradation, which subsequently activated glycolytic flux and promoted cell cycle progression. LncRNA OCC-1 exerted its function by binding to and destabilizing HuR (ELAVL1), which inhibited the cell cycle transition in colorectal cancer. LncRNA LINRIS, on the other hand, blocked the degradation of IGF2BP2 through the ubiquitination-autophagy pathway. LncRNA uc.134 repressed hepatocellular carcinoma progression by inhibiting the CUL4A-mediated ubiquitination of LATS1 and increasing YAP^S127^ phosphorylation. LncRNA CRNDE directly bound to the splicing protein SRSF6 to reduce its protein stability and thus regulated alternative splicing (AS) events [24–29]. Nevertheless, the function of lncRNAs in the UPS system is mainly performed as molecular scaffolds to mediate the masking of protein ubiquitination sites or promote the binding of ubiquitin ligase to substrates. Whether there are other mechanisms involved still requires further investigation.

In this study, we identified a novel MDH2-interacting lncRNA renamed malate dehydrogenase degradation helper (MDHDH). The lncRNA MDHDH is characterized by a full length of 749 nt and is located at chromosome X. When the expression level of MDHDH was artificially upregulated in glioma cell lines, we observed significant remodeling of NAD+ metabolism as well as enhanced MDH2 degradation. As the first lncRNA that was shown to directly bind to and regulate MDH2, MDHDH mediated the interaction of MDH2 and PSMA1. PSMA1 subsequently promoted the proteasomal degradation of ubiquitinated MDH2, thus inhibiting the glycolytic process, affecting the NAD+/NADH ratio, regulating the AMPK/mTOR pathway and promoting autophagy as well as cell apoptosis. We also demonstrated the upstream PRC2/EZH2-induced epigenetic silencing pattern of MDHDH, which indicated that the PRC2 inhibitor GSK126 has potential therapeutic value for GBM treatment. Clinically, high MDHDH expression levels were negatively correlated with the WHO grades of gliomas and positively associated with survival (overall and disease-free) of glioma (LGG and GBM) patients, further suggesting that MDHDH might be a potential biomarker and therapeutic target for glioma patients.

## Methods and materials

### Clinical sample preparation and high-throughput sequencing analysis

A total of 144 glioblastoma samples and 8 normal brain samples (normal cortical brain tissue obtained during glioma surgery) were collected from the Department of Neurosurgery of Shandong Provincial Hospital affiliated with Shandong University. The research was approved by the Research and Ethics Committee of Shandong Provincial Hospital.

To detect the candidate lncRNAs in human GBM tissues, total RNA was first extracted with TRIzol Reagent (Invitrogen, CA, USA) from the tissues of five GBM patients (five GBM samples, four paracancerous samples and five normal brain samples in total). Labeling and hybridization to the Affymetrix GeneChip Human Exon Arrays-Gminix- lncRNA-WT (Gminix, Shanghai, China) based on extracted RNA were then conducted. The lncRNAs were carefully constructed using publicly available transcriptome databases (RefSeq, UCSC Known Genes, Ensemble, NONCODE, etc.). After quantile normalization of the raw signal, 249 lncRNAs with significant differences among GBM, paracancerous and normal brain tissues (FDR<0.01) were chosen for further data analysis. For RNA-seq analysis, mRNA sequencing was conducted using the Illumina HiSeq Platform (PE150). Briefly, total RNA was isolated and subjected to cDNA library construction, in which the clean (high-quality) reads were aligned to the Human Genome Reference (GRCh38). Gene expression normalization was performed by Fragments per Kilobase per Million Mapped Fragments (FPKM). Using a high-throughput sequencing technique (Affymetrix GeneChip® Human Transcriptome Array 2.0), we defined clusters of differentially expressed lncRNAs in glioma specimens and paracancerous tissues. The suffix E-Signal represents the paracancerous tissues, and the suffix T-Signal represents the tumor tissues. Clusters in green indicate the downregulated lncRNAs, and clusters in red indicate the opposite.

### Cell culture and reagents

The high-grade human glioma cell lines U251 and U87 were obtained from American Type Culture Collection (Manassas, VA, USA) and used for in vitro experiments. The normal human astrocyte (NHA or HA) cell line was obtained from ScienCell (Carlsbad, CA, USA). Tumor cells were maintained as monolayer cultures in Dulbecco’s modified Eagle’s medium (DMEM) supplemented with 10% fetal bovine serum (FBS), 100 units/mL penicillin and 100 μg/mL streptomycin. The culturing environment was 37 °C with 5% CO_2_. GSC_U251_ (Glioma stem cell derived from U251) were obtained from the U251 cells cultured in DMEM/F12 medium supplemented with 2% B27, 25ng/ml human recombinant basic fibroblast growth factor, 25ng/ml epidermal growth factor and 1% penicillin/streptomycin. Half of the sphere-forming medium was replaced every other day.

### Reagents, antibodies and MDHDH sequence information

Detailed information on the specific reagents and antibodies is listed in Supplementary Table 1. LncRNA MDHDH reference sequence information is listed in Supplementary Table 2, and NCBI-ORFinder potential peptide alignment results are listed in Supplementary Table 3.

### RNA chromogenic in situ hybridization (CISH) and RNA fluorescence in situ hybridization (FISH) assay

To detect the expression status of MDHDH in different grades of glioma, a tissue microarray obtained from Outdo Biotech Co., Ltd. (S/N: HBraG090PG01-M-081, WHO grade 1-4 and normal brain tissue) was employed. The MDHDH-CISH probes were designed and synthesized by Boster Biological Technology Co., Ltd. The CISH assay kit was obtained from the same company and applied according to the manufacturer’s protocol.

In addition, to detect the subcellular localization of lncRNA MDHDH, a Fluorescence in Situ Hybridization Kit (RiboBio, Guangzhou, China, FISH probe sequences listed in Supplementary Table 4) was utilized. The probes were designed and synthesized by RiboBio, Guangzhou. U87 and U251 cells were used in the experiments.

### Transfection of the MDHDH smart silencer

RiboBio lncRNA Smart Silencer was provided by Guangzhou RiboBio Co., Ltd. (the smart silencer sequences are listed in Supplementary Table 4). Transfection was conducted according to the manufacturer’s protocol. Briefly, cells were grown to 30-50% confluence and transfected with oligonucleotides mixed with Lipofectamine 2000 at a concentration of 100 nM. The smarter silencer was represented as the pool containing three siRNAs and three antisense oligonucleotides that target different sites of MDHDH.

### Vector construction and small interfering RNA (siRNA)

To generate a GBM cell model that is close to the basal MDHDH expression level of normal brain tissue, we synthesized the MDHDH sequence (NR_028345) via Biosune (Shanghai, China) and cloned it into the eukaryotic expression vector (pcDNA3.1) (Invitrogen, CA, USA). This vector possesses the restriction endonuclease site XhoI, which can be used to generate RNA probes for the subsequent RNA pull-down experiments.

We obtained the three siRNAs of PSMA1 from GeneralBiol (Anhui, China). To confirm whether the molecular scaffold function and phenotypic regulation function of MDHDH are dependent on PSMA1, siRNA (5’-GGGCAGGAUUCAUCAAAUUTTAAUUUGAUGAAUCCUGCCCTT-3’) duplexes with proven knockout efficiency and targeting human PSMA1 (PSMA1 siRNA) were used to transfect GBM cells. The siRNA transfection was performed using the transfection reagent Lipofectamine RNAiMAX (Invitrogen, CA, USA) according to the manufacturer’s protocol. Briefly, the siRNA was added to each well at a final concentration of 100 nM. Six hours later, the medium was replaced with DMEM containing 10% FBS, and the cells were incubated for 72 h. The PSMA1 expression levels were determined by qRT–PCR and Western blotting.

### Quantitative reverse-transcription polymerase chain reaction (qRT–PCR)

Briefly, total RNA was extracted from the cell lysates, cytoplasm extracts or nuclear extracts by TransZol Up (Transgen, Beijing, China). RNA was quantitatively analyzed using a Nanodrop (Nanodrop Technologies, Rockland, DE, USA). Total RNA (1 μg) was reverse transcribed into cDNA using a cDNA synthesis kit (Transgen, Beijing, China) according to the manufacturer’s instructions. RT–PCR was performed in a Bio-Rad CFX connect real-time system detector with Transgen SYBR Green Supermix. The reactions were analyzed using Bio-Rad CFX Maestro software (Version 4.1). The threshold cycles (CT) were calculated, and the relative gene expression was analyzed after normalizing to beta-actin. The experimental primers were designed and produced by Takara (Japan) and are listed in Supplementary Table 5.

### Cell viability assay

The Cell Counting Kit-8 (CCK-8) (Dojindo Molecular Technologies, Inc. Beijing, China) was used to determine cell viability. After overexpression or RNA silencing, cells were seeded at 1×10^3^ cells/well in 96-well plates. At different time points, the culture medium was replaced with 100 μL of fresh medium containing 10 μL of CCK-8 solution. The cells were further incubated for 2 h at 37 °C , and the optical density (OD) at 450 nm was measured. Each experiment was repeated three times.

### Wound-healing and transwell assays

A wound healing assay was performed in 6-well cell culture plates (Corning, USA). The scratching step was performed vertically on the center of each well using 200 μL pipettes, and the scratched cells were cultured for 12 h and 24 h in high-glucose DMEM without fetal bovine serum (FBS). Images were obtained using a microscope at 200×, and the gap distance was measured by the plotting scale of the software. The proportion of changes was calculated and statistically analyzed. Transwell assays were applied to evaluate the invasion and migration capacities. Glioma cells were seeded in transwell chambers (24-well format; Corning, USA). For the invasion test, we used Matrigel-coated chambers (BD Biosciences, NJ, USA). Cells were cultured with 100 μL serum-free DMEM in the upper chamber for 6 h, and the lower chamber containing 500 μL medium was supplemented with 20% FBS for transwell tendency. Then, the cells in the upper transwell chambers’ supernatant and attachments were removed. Cells on the bottom surface of the chambers were fixed in methanol for 5 min, stained with crystal violet (Solarbio, Beijing, China) and counted in three random fields under a microscope. Each experiment was repeated three times.

### Western blotting

In brief, the cells were harvested and lysed with protein extraction agent (RIPA, Solarbio, Beijing, China). Considering the specific application, a Nucleus and Cytoplasmic Protein Extraction Kit (Beyotime, Shanghai, China) was utilized. A total of 25-50 μg of protein per sample per lane was mixed with loading buffer (Epizyme, Shanghai, China) and loaded for sodium dodecyl sulfate–polyacrylamide gel electrophoresis (10%-12.5% SDS–PAGE gel preparation kit, Epizyme, Shanghai, China). Primary antibodies were incubated overnight at 4 °C . Secondary antibodies (goat anti-rabbit and mouse IgG-HRP, Abmart, Shanghai, China) were incubated for 1-2 h at RT. The proteins were visualized using chemiluminescence (ECL) (Affinity Biosciences, OH, USA) and a detection system (Tanon 4800, Tanon, Shanghai, China). All primary and secondary antibody information is listed in Additional file 1.

### RNA segmentation, biotin-labeled RNA pulldown and mass spectrometry (MS) assay

Human MDHDH cDNAs (sense and antisense; Biosune Biotech, Shanghai, China) and truncated constructs were transcribed in vitro using the MEGAscript T7 Kit (Thermo Fisher Scientific, MA, USA). The full-length transcript of MDHDH is 746 nt in length; Δ1, Δ2, Δ3, Δ4, Δ5, Δ6, Δ1-del, Δ2-del and Δ5-del correspond to nt 1–293, 294–564, 565–749, 1-49/495-577, 50-494, 578-749, 1-47/181-293, 390-564 and 181-293/390-494 of MDHDH, respectively. For plasmid extraction, an OMEGA Endo-Free Plasmid Mini Kit II (OMEGA BioTek, Guangzhou, China) was applied. For restriction enzyme digestion, FastDigest XhoI (Thermo Fisher Scientific, MA, USA) was utilized. Agarose gel electrophoresis images were obtained by Quantity One software. For DNA purification, we utilized a TIANquick Mini Purification Kit (Tiangen Biotech, Beijing, China). The 3’ ends of the resultant transcripts were labeled with biotin using the Pierce™ RNA 3’ End Desthiobiotinylation Kit (Thermo Fisher Scientific, MA, USA) to generate RNA probes for RNA pulldown, which was performed using the Pierce™ Magnetic RNA–Protein Pull-Down Kit (Thermo Fisher Scientific, MA, USA) following the manufacturers’ guidelines. Eluted proteins were detected by mass spectrometry analysis (MS). MS was utilized to identify the proteins that interacted with full-length and/or truncated RNA probes. The filter-aided sample preparation (FASP) method was used for sample preparation [30]. Proteins were digested with trypsin (0.4 μg/μL, Promega) overnight. Peptides were desalted and concentrated using C18-based solid phase extraction prior to analysis by high resolution/high mass accuracy reversed-phase (C18) nano-LC–MS/MS. All raw files were processed using Proteindiscover software (version 1.4, Thermo Scientific) for database searching. MS/MS spectra were searched in the UniProtKB/Swiss-Prot human database. MS analysis was performed by the Advanced Medical Research Institute of Shandong University, and the protein list obtained from the RNA pulldown assay is shown in Supplementary Table 12. After PPI (protein–protein interaction) and gene or pathway enrichment (MCODE) analyses [31], proteins that may interact with RNA were verified by Western blot.

### RIP, Co-IP and ChIP Assays

U87 and U251 cells were seeded, transfected and lysed with lysis buffer (RNase inhibitor included) using an RNA immunoprecipitation Kit (Geneseed, Guangzhou, China), producing a mixture containing bait-target complexes and other irrelevant proteins. Then, a capture antibody and protein A/G magnetic beads were added to the mixture to specifically bind bait protein after antibody-bead crosslinking. According to the manufacturer's instructions, two kinds of columns were utilized to harvest the RNA and proteins bound to magnetic beads. For RIP-seq analysis, RNA sequencing was conducted by the application of Illumina HiSeq Platform (PE150). Briefly, total MDH2-interacting RNAs were isolated and subjected to a library construction, in which the clean (high-quality) reads were aligned. All identified lncRNAs referenced in Supplementary Table 16. Meanwhile, MDHDH-specific primers were used to conduct qRT–PCR analysis on the retrieved RNA. We detected both total RNA (input controls) as well as normal mouse IgG controls to verify that the previously detected signals specifically originated from the MDH2- or PSMA1-binding RNAs. The eluted protein sample (eluted from anti-PSMA1, anti-MDH2 and mouse IgG antibodies) was added to the loading buffer and heated at 95 °C for 15 min for SDS–PAGE analysis. Anti-MDH2 or anti-PSMA1 primary antibodies were used to incubate the transferred PVDF membrane and detect whether the binding degree of PSMA1 and MDH2 changes with the expression status of MDHDH.

U87 and U251 cells were grown to 80% confluence. ChIP assays were performed with a SimpleChIP Enzymatic Chromatin IP kit (Cell Signaling Technology, #9003 MA, USA) according to the manufacturer’s directions. Native chromatin immunoprecipitation was performed with an anti-H3K27me3 antibody (Cell Signaling Technology, #9733, 1:50) or anti-H3 (Cell Signaling Technology, #4620, 1:50). Rabbit IgG was employed (ABclonal, AC005, 5 μg) as a negative control. qPCR analysis was performed to detect the DNA fragments immunoprecipitated with H3K27me3. The primer pairs are listed in Supplementary Table 5.

### Cell bioenergy tests

Transfected U87 and U251 cells were seeded in XFe 96-well microplates (5×10^3^ cells per well) (Agilent Technologies, Sana Clara, USA) for 24 h. Cells were rinsed and incubated in base medium (Agilent Technologies) at 37 °C for 1 h. The extracellular acidification rate (ECAR) and oxygen consumption rate (OCR) were measured in real time with a Glycolysis Stress Test Kit and Mito Stress Test Kit, respectively, using a Seahorse XFe96 Analyzer (Agilent Technologies) following the manufacturer’s instructions. Data were normalized by the cell number.

### Transfection with adenovirus expressing the mCherry-GFP-LC3B fusion protein

To detect changes in autophagic flux, an adenovirus expressing the mCherry-GFP-LC3B fusion protein (Ad-mCherry-GFP-LC3B) was obtained from Beyotime, China. Cells grown to approximately 70% confluence were transfected with Ad-mCherry-GFP-LC3B according to the manufacturer’s instructions. Ad-mCherry-GFP-LC3B–transduced glioma cells were transfected with different plasmid constructs, visualized by fluorescence microscopy and quantified with FIJI software. DMEM high-glucose medium (with FBS) was replaced with Earle's Balanced Salt Solution (with Ca^2+^& Mg^2+^) (EBSS) to induce GBM cell autophagy as a positive control. When autophagosomes accumulated, both red and green LC-3 puncta were observed. The merging of the two channel images generated a yellow signal, which indicated that the autophagy process was not completed (autophagosomes had not yet fused with lysosomes to form autolysosomes). The change in fluorescence signal represented the formation of autophagosomes and the process of autophagosome-lysosome association (yellow- no autophagy; green puncta- autophagosome formation; red puncta- autophagy lysosome formation).

### Mitochondrial membrane potential (ΔΨm) assay

A JC-1 probe was employed to measure mitochondrial depolarization in GBM cell lines. Briefly, cells cultured in six-well plates after the indicated treatments were incubated with an equal volume of JC-1 staining solution (5 pg/ml) at 37 °C for 20 min and rinsed twice with PBS. The mitochondrial membrane potential was monitored by determining the relative amounts of dual emissions from mitochondrial JC-1 monomers or aggregates using an Olympus fluorescence microscope. Mitochondrial depolarization is indicated by an increase in the green/red fluorescence intensity ratio.

### NAD+/NADH measurement

Transfected U87 and U251 cells were collected to determine the NAD+ levels using an NAD+/NADH assay kit with WST-8 (Beyotime, Shanghai, China) and a Mitochondria/Cytosol Fractionation Kit (BioVision, K256-100 CA, USA) according to the manufacturer’s instructions. In brief, cells (1×10^6^/sample) were lysed and isolated with a Mitochondria/Cytosol Fractionation Kit. To measure the total NAD+/NADH ([NAD_total_]), 20 μL of cyto-lysates was added to a 96-well plate. To measure NADH ([NADH]), the lysed cells were incubated at 60 °C for 30 min, and 20 μL was added to a 96-well plate. Subsequently, 90 μL of alcohol dehydrogenase was added and incubated at 37 °C for 10 min. Finally, 10 μL of chromogenic solution was added to the plate, and the mixture was incubated at 37 °C for 10 min. A standard curve was generated and measured at the same time as the samples. The absorbance values were measured at 450 nm and analyzed on a plate reader. The amount of NAD+ was derived by subtracting NADH from the total NAD+/NADH ([NAD+] = [NAD_total_] - [NADH]; [NAD+]/[NADH] = ([NAD_total_] - [NADH])/[NADH]).

### Extracellular pyruvate and lactic acid assays

For the pyruvate assay, we utilized a pyruvate testing kit (Jiancheng Bioengineering Institute, Jiangsu, China). We mixed 100 μL of the specimen with 1 mL of the assay reagent in the kit, added 100 μL of the standard pyruvate (0.2 μmol/ml) and 100 μL of double-distilled water to 1 mL of the assay reagent, reacted in a water bath at 37 °C for 10 minutes, and measured the absorbance value at 505 nm. The result was normalized to the protein concentrations of the samples, which were determined using the BCA protein Assay Kit (Vazyme Biotech, Jiangsu, China). For the lactate assay, we utilized a lactic acid testing kit (Jiancheng Bioengineering Institute, Jiangsu, China). This kit applies NAD+ as the hydrogen acceptor. LDH can catalyze the dehydrogenation of lactic acid to produce pyruvate, which converts NAD+ into NADH. Insoluble blue–purple formazan can be produced by dehydrogenases, which originate from the interactions of nitroblue tetrazolium (NBT) and NADPH in the presence of phenazine methosulfate (PMS )[32]. The absorbance at 530 nm is linearly related to the content of lactic acid. Then, 20 μL supernatant samples (DMEM with no phenol red) were mixed with 200 μL NBT solution and 1 mL enzyme reaction buffer. After 10 min at 37 °C, stop buffer was added, and the absorbance values (530 nm) were monitored. The lactic acid concentrations of the samples were calculated using the difference in two absorbance values at 540 nm and the lactic acid standard. The result was normalized to the protein concentrations of the samples, which were determined using the BCA protein Assay Kit (Vazyme Biotech, Jiangsu, China).

### In vivo tumor formation assay

Aiming at the target lncRNA MDHDH, we designed a lentivirus expression vector using the human eukaryotic translation elongation Factor 1 α1 promoter. GL261 cells, U87 cells, C57 cells and BALB/c nude mice were chosen for the in vivo experiments. After lentivirus infection and puromycin selection, we set up the experimental cell line LV-EF1a>MDHDH-CMV>Luciferase/T2A/Puro (Cyagen, Guangzhou, China) and the control cell line LV-CMV>Luciferase/T2A/Puro (Cyagen, Guangzhou, China) for animal experiments. Four-week-old male C57 mice were purchased from Vital River Laboratories (Beijing, China). The mice were assigned randomly to two groups (n=10 each group). GL261 cells were subcutaneously injected into the right frontal lobes of the mice. The animals were anesthetized and dissected 3 weeks later. Tumor volumes were monitored by bioluminescence (IVIS Spectrum in vivo imaging system, PerkinElmer, MA, USA).

In addition, the U87 cells were injected at the same position mentioned above into three groups (n=10 each group) of four-week-old male Nu/Nu mice (Vital River Laboratories, Beijing, China). The Nu/Nu mice were anesthetized and dissected 3 weeks later.

For MDHDH overexpression with PSMA1 knockdown U87 cell line (OE MDHDH+shPSMA1), the following lentiviral expression vectors were applied: LV-U6>PSMA1-shRNA>SV40/BSDr (Origene, Jiangsu, China)

Sense sequence: GCCTGTGTCTCGTCTTGTATC

Top Strand (5'-3'): CACCGCCTGTGTCTCGTCTTGTATCTTCAAGAGAGATACAAGACGAGACACAGGC

Bottom Strand (5'-3') AAAAGCCTGTGTCTCGTCTTGTATCTCTCTTGAAGATACAAGACGAGACACAGGC

Target (3'-5'): CGGACACAGAGCAGAACATAGAAGTTCTCTCTATGTTCTGCTCTGTGTCCGAAAA

After lentivirus infection and puromycin/blasticidin double selection, the OE MDHDH+shPSMA1 U87 cell line was obtained. The same procedure as above was used for tumor formation and bioluminescence experiments.

### Immunohistochemistry staining

Tumor tissues were paraformaldehyde fixed, paraffin embedded, sectioned (5 μm) and transferred onto glass slides. The deparaffinized sections were incubated in H_2_O_2_ for 10 min and rehydrated in a series of ethanol solutions. Ki67 or MDH2 staining was performed after antigen retrieval with 1 mM EDTA (pH 8), 10 mM citrate buffer, or 1 mM EDTA plus 10 mM Tris-Cl (pH 8). Sections were washed 3 times in PBS, treated with 3% H_2_O_2_ in PBS for 15 minutes, blocked in 10% goat serum and 0.3% Triton X-100 in PBS for 1 hour, and incubated with primary antibody against MDH2 or Ki67 overnight. Secondary antibody (ZSGB-BIO, Beijing, China) was applied for 30 min at 37 °C. The sections were developed with the Polink-2 plus® Polymer HRP Detection System (ZSGB-BIO, Beijing, China) according to the manufacturer’s protocol. Next, the sections were visualized by using a diaminobenzidine (DAB) substrate kit (ZSGB-BIO, Beijing, China) for 10 min. After intensive washing, the sections were counterstained with hematoxylin, dehydrated and cover-slipped. Then, the samples were observed with an Olympus BX41 microscope and an Olympus DP72 camera. Data were analyzed with FIJI software (based on ImageJ version 1.52). The intensity score was assessed as 0 (negative), 1 (weak), 2 (moderate), and 3 (strong). The IHC score (histochemistry score, H-score) = (percentage of weak intensity area ×1) + (percentage of moderate intensity area ×2) + (percentage of strong intensity area ×3).

### Online tools, Datasets and software

Cistrome Data browser (T98G cell line, CistromeDB:103264, Normal brain tissue, CistromeDB:6651) [33];

GEPIA (Gene Expression Profiling Interactive Analysis) 1 and 2 (http://gepia.cancer-pku.cn/andhttp://gepia2.cancer-pku.cn/#index) [34];

CGGA (Chinese Glioma Genome Atlas) dataset (http://www.cgga.org.cn/) [35];

Metascape online tools (https://metascape.org/) [31] for pathway enrichment and MCODE (Molecular Complex Detection) analysis;

GTEx (Genotype-Tissue Expression) dataset;

GEO dataset: GSE60666 [36]; GSM772772 [37]; GSM3061513 [38];

R software (version 1.2.1335) and the ggplot and pROC [39] packages.

### Statistical analysis

Statistical analysis was performed using Prism 7 software (GraphPad Software, CA, USA). Student’s t test was used to compare differences between two groups. Survival curves were plotted and analyzed using the Kaplan–Meier method and log-rank test. All data were displayed as the mean ± standard error of the mean (SEM), and a *P* value < 0.05 was considered to be statistically significant.

## Results

### Screening and identification of MDH2-interacting lncRNAs

**Fig. 1:**
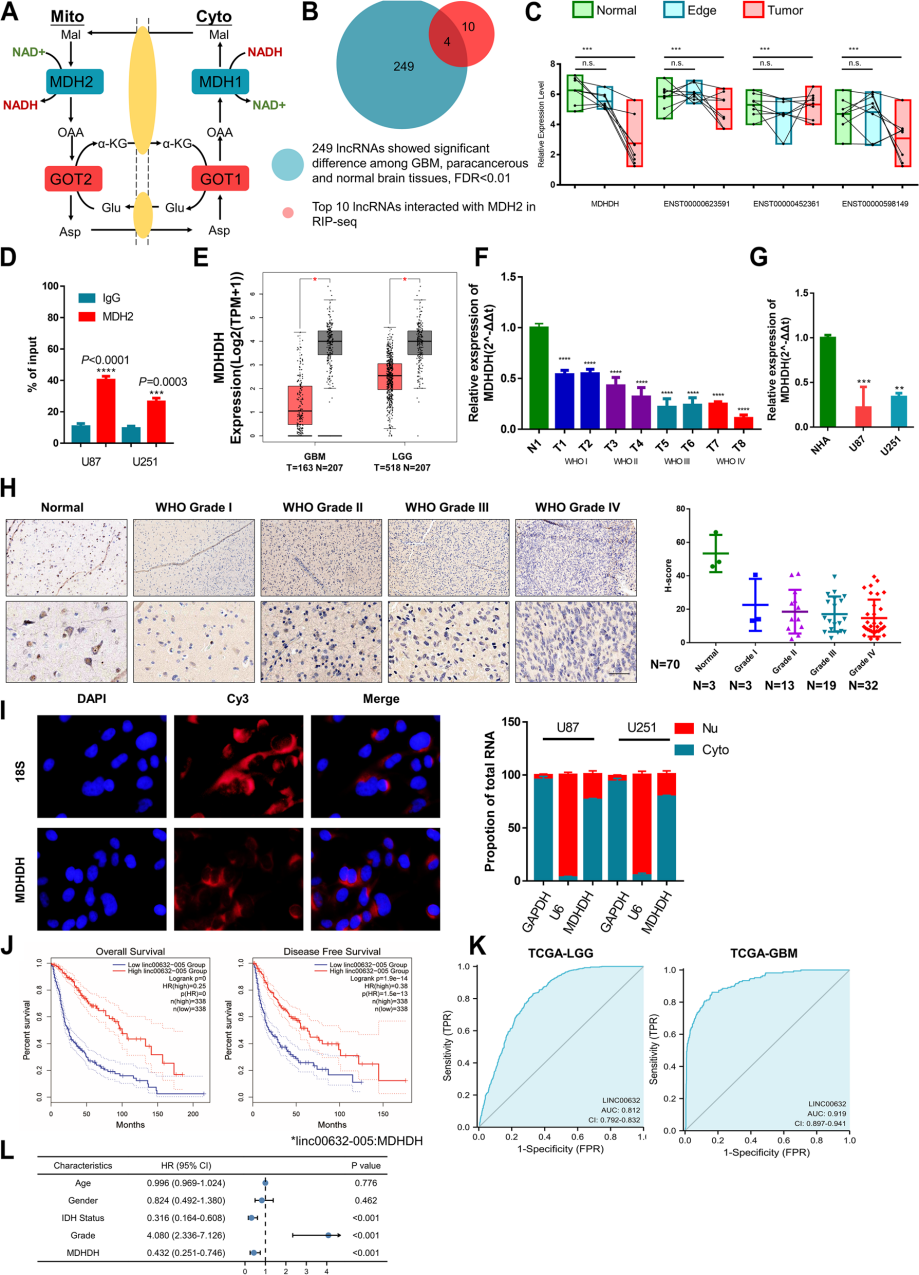
LncRNA MDHDH was significantly downregulated in gliomas. LncRNA profiling and public datasets reveal MDHDH is a candidate glioblastoma multiforme suppressor. **A**, Graphical representation of the malate–aspartate shuttle. As shown, this shuttle is operated by two pairs of enzymes, cytosolic GOT1 and MDH1 as well as mitochondrial GOT2 and MDH2, which act in concert to transfer reducing equivalents across the mitochondrial membrane. **B** A screening strategy was used to identify key MDH2-binding lncRNAs in GBM. RIP-seq experiments were performed to identify the top ten MDH2-binding lncRNAs. The top ten and high-throughput differentially expressed lncRNAs were intersected to obtain 4 transcripts (ENST00000623591, ENST00000452361, ENST00000598149 and MDHDH). **C** Box-plot analysis of MDH2-binding lncRNAs (corresponding to Figure 1B) in the microarrays (green: normal brain tissue; blue: tumor margin; red: tumor tissue). **D** RNA immunoprecipitation using MDH2 antibody (IgG as control) to capture RNA from glioma cell lines (U87 and U251). n=3 independent experiments, two-tailed Student’s t test. **E** Graphic representation of the relative MDHDH expression level (Log2(TPM+1)) in different tissues (GBM vs. GTEx, LGG vs. GTEx). GBM and LGG represent glioblastoma multiforme (GBM, WHO grade IV) and low-grade gliomas (LGG. WHO grade II and III) in TCGA datasets. GTEx represents normal brain tissue in the GTEx database (*, statistically significant). **F** The differential expression levels of MDHDH in the clinical glioma specimens classified into World Health Organization (WHO) grades were examined using qRT–PCR. The results are presented as the mean ± s.d. from three independent experiments. **G** The differential expression levels of MDHDH in the normal human astrocyte (NHA) cell line and GBM cell lines (U87 and U251). The results are presented as the mean ± s.d. from three independent experiments. **H** Representative RNA chromogenic in situ hybridization (CISH) images of MDHDH probe staining in human glioma tissues (WHO grade I-IV) and human normal brain tissue (upper). The CISH probe appeared brown (positive) after binding to target RNA (MDHDH). Scale bar = 100 μm. MDHDH H-score of different glioma tissues (WHO I-IV) and human normal brain tissue (right panel). **I** RNA fluorescence in situ hybridization (FISH) to detect Cy3-MDHDH (red) in U87 cells. Nuclei were stained with DAPI (blue), and images were merged. qRT–PCR for MDHDH expression in the cytoplasmic and nuclear RNAs isolated from NHA, U87 and U251 cells. Scale bar = 10 μm (n.s., not significant; *, *P*<0.05; **, *P*<0.01; ***, *P*<0.001). **J** Assessment of the overall survival (OS) and disease-free survival (DFS) of glioma patients with low or high LINC00632-005 (MDHDH) expression levels (cutoff value: 50%). **K** ROC curve analyses to evaluate the diagnostic potential of LINC00632 for GBM and LGG. **L** Univariate analysis of 144 glioma patients using the Cox regression model. The HR (hazard ratio) for MDHDH was 0.432 (0.251-0.746), a protective factor for the disease

To identify potential targets related to NAD+ metabolism in gliomas (Fig. 1A), RNA immunoprecipitation-sequencing (RIP-seq) was used to identify MDH2-interacting lncRNAs. The top ten MDH2-binding lncRNAs (fold change > 2) were then integrated with the differentially expressed lncRNAs among the normal/paracancerous/GBM tissues. (FDR < 0.01) (Fig. 1B). Four candidate lncRNAs were ultimately identified, of which NONHSAT138800 (NONCODE TRANSCRIPT ID NONHSAT138800), renamed MDHDH (malate dehydrogenase degradation helper), exhibited a significant downregulation pattern in glioma tissues compared to paracancerous and normal brain tissues (Fig. 1C). Moreover, RIP assays further confirmed the significant enrichment of MDHDH by MDH2 compared to the IgG control (Fig. 1D).

MDHDH is one of the transcript variants of LINC00632 and has different annotations in multiple databases (NONCODE annotation ID: NONHSAT138800.2, NCBI Reference Sequence: NR_028345; LINC00632-005/205). MDHDH is characterized by a full length of 749 nt (Supplementary Table 2) and is located on chromosome X. We used the NCBI ORFinder online tool (https://www.ncbi.nlm.nih.gov/orffinder/) to detect the number of ORFs in the RNA sequence. The results show 14 sense ORFs with the potential to encode peptides. However, these 14 peptides indicated no significantly similar proteins or peptides when they were compared with the Swiss-Prot database using the NCBI BLAST tool (Supplementary Table 3). Additionally, the PhyloCSF value of MDHDH, which was calculated to verify the conservation of the sequence, was negative (Supplementary Figure S1A). In conclusion, we identified MDHDH as a long noncoding RNA with no protein encoding potential.

As a type of intergenic lncRNA, LINC00632 has significant tissue specificity (Supplementary Figure S1C-E). Interestingly, the expression levels of all LINC00632 transcript variants were reduced in both GBM and LGG (Fig. 1E and Supplementary Figure S1E), among which MDHDH was one of the most significantly downregulated transcripts (Supplementary Figure S1F). Subsequent qRT–PCR analyses in a series of 144 clinical samples (Fig. 1F), GBM cell lines and the NHA cell line (Fig. 1G) confirmed this result. The status of MDHDH was additionally evaluated by chromogenic in situ hybridization (CISH). The results of the tissue microarray showed that the expression levels of MDHDH were indeed negatively correlated with the pathological grades of gliomas (Fig. 1H). Fluorescence in situ hybridization (FISH) assays and subcellular fractionation assays were further utilized to examine the distribution of MDHDH in U87 cells. The results show that MDHDH was mainly located in the cytoplasm of the cells (Fig. 1I). Collectively, these results indicate that MDHDH might be involved in NAD+ metabolism and the progression of GBM.

### Public datasets reveal LINC00632 and its transcript variant MDHDH as potential glioma suppressors

Clinicopathological and genetic characteristics are associated with overall survival in glioma patients. Patient age and genetic features, including codeletion of 1p/19q and IDH mutations, have been reported to be associated with a favorable prognosis. We therefore analyzed whether the high or low expression of LINC00632 was correlated with any of these characteristics. Among the panglioma datasets of the TCGA database (TCGA-GBM and TCGA-LGG, n=696), LINC00632 was statistically associated with patient age (*P* < 0.001), codeletion of 1p/19q (*P* < 0.001) and IDH mutations (*P* < 0.001) (Supplementary Table 7). Univariate logistic regression of LINC00632 expression also provided consistent results (Supplementary Table 8). When panglioma patients were grouped based on the WHO grades, 1p/19q codeletion, IDH mutation, primary treatment outcome and survival events (PFI, DSS, OS), the expressional difference in LINC00632 between each group was statistically significant (Supplementary Figure S2). Moreover, LINC00632 was validated as an independent prognostic indicator in univariate and multivariate Cox regression analyses of overall survival (OS) [hazard ratio (HR) = 0.406, 95% confidence interval (CI) = 0.315 to 0.524, *P* < 0.001 (univariate analysis); HR = 0.713, 95% CI = 0.531-0.958, *P* = 0.025 (multivariate analysis) Supplementary Table 9], disease-specific survival (DSS) [HR = 0.396, 95% CI = 0.302 to 0.520, *P* < 0.001 (univariate analysis); HR = 0.677, 95% CI = 0.493 to 0.928, *P* = 0.015 (multivariate analysis), Supplementary Table 10] and progression-free interval (PFI) [HR = 0.449, 95% CI = 0.361 to 0.560, *P* < 0.00 1 (univariate analysis); HR = 0.647, 95% CI = 0.501 to 0.836, *P* < 0.001 (multivariate analysis), Supplementary Table 11] in panglioma patients.

To estimate the epidemiological value of MDHDH in glioma patients, we divided 776 patients from the TCGA glioma datasets into two groups according to the relative expression levels (cutoff value: 50%) of MDHDH (TCGA transcript annotation: LINC00632-005). The OS and disease-free survival (DFS) of patients with LGGs and GBMs with lower vs. higher MDHDH expression were estimated using Kaplan–Meier curves. The results show that the upregulated level of MDHDH was correlated with longer OS and DFS in GBM and LGG patients, respectively (Fig. 1J). Receiver operating characteristic (ROC) curve analyses were subsequently performed to evaluate the diagnostic potential of MDHDH (Fig. 1K). The results also indicate that MDHDH could be used to clearly distinguish between patients with gliomas and healthy controls (ROC baseline data shown in Supplementary Table 6). High diagnostic accuracy was observed for diagnosing GBM and LGG, as measured by the AUC (GBM group: 0.919 LGG group: 0.812). In addition, multivariate Cox analysis in 144 clinical specimen cohorts indicate that MDHDH was an independent prognostic factor (Fig. 1L).

In summary, these data suggest that in panglioma patients, LINC00632 and its most downregulated transcript variant, MDHDH, might serve as novel diagnostic markers, prognostic indicators and candidates for GBM suppressors.

### Overexpression of MDHDH inhibited the malignant phenotypes of GBM cells

**Fig. 2:**
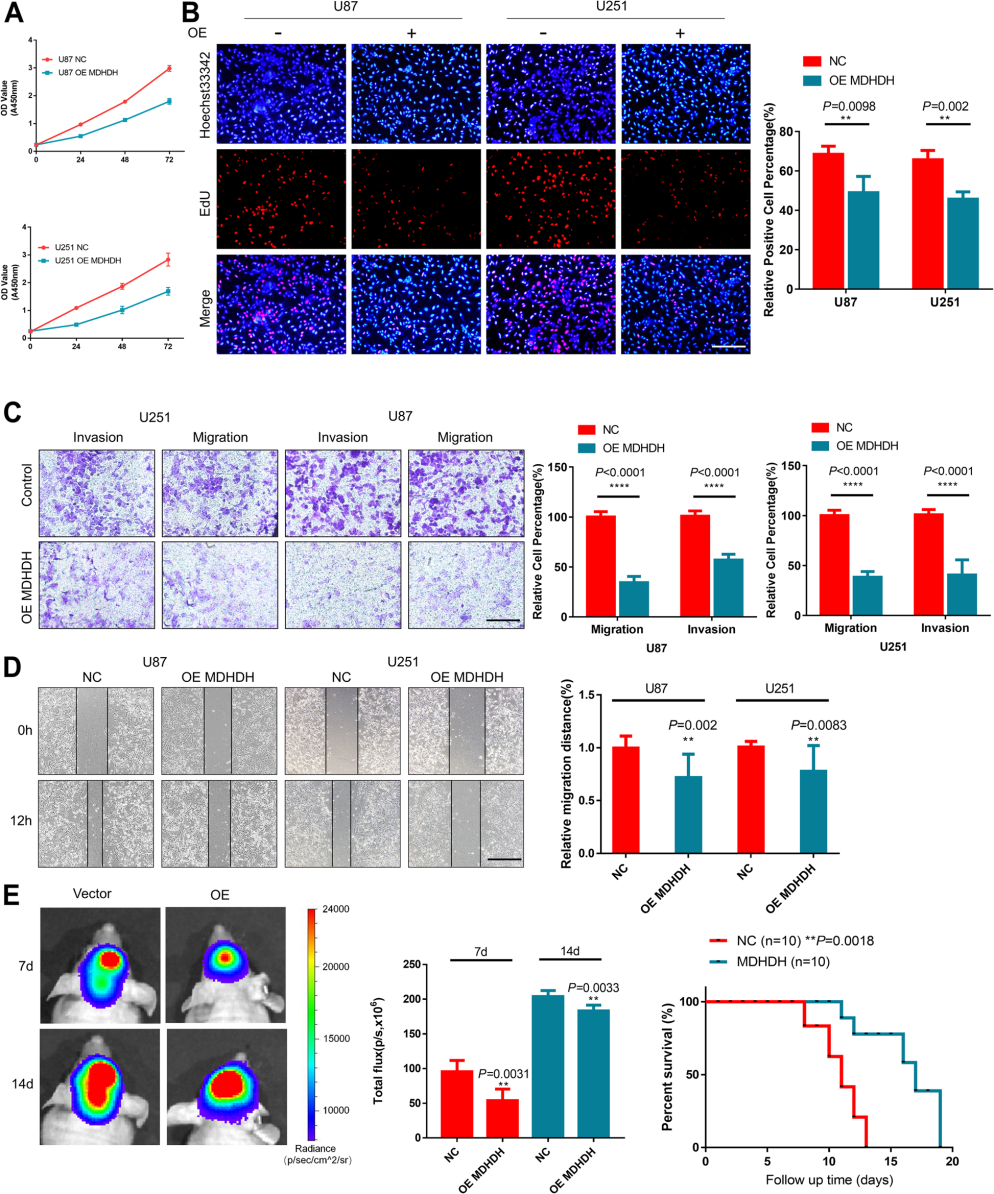
MDHDH inhibited the malignant phenotypes of GBM cells in vitro and in vivo. **A** U87 and U251 cells were transfected with the indicated plasmid constructs, and GBM cell line viability was examined by CCK-8 assay (left panel: U87 cell line, right panel: U251 cell line). **B** U87 and U251 cell proliferation was determined by an EdU staining assay. Positive cells in 5 random fields were counted (Student’s t test; scale bar = 200 μm). **C** Migration and invasion of the transfected U87 and U251 cell lines (NC and OE MDHDH) were determined by transwell assay. Stained images were counted after 12 h of seeding (Student’s t test; scale bar = 100 μm; column means of triplicate assays). **D** Cell migratory capability assessed by wound healing assay in GBM cell lines transfected with OE-MDHDH or control vector (as a negative control). Phase-contrast images were acquired at 12 h after scratching. The corresponding data relative to the indicated time courses are shown in the graph (right panel, Student’s t test; scale bar = 200 μm; column means of triplicate assays). **E** Representative images and total flux of nude mice 7 days and 14 days after intracranial implantation of the luciferase-tagged U87 cells transfected with MDHDH or control plasmid by IVIS spectrum. The survival curves of the nude mice with intracranially xenografted tumors were recorded for 15 days. (n.s., not significant; *, *P*<0.05; **, *P*<0.01; ***, *P*<0.001)

To elucidate the functions of MDHDH in gliomas, we transfected both U87 and U251 glioma cells with plasmids expressing MDHDH (pcDNA3.1, OE MDHDH, Supplementary Figure S3A) or MDHDH Smart Silencer (siMDHDH). Nonspecific vectors and scramble siRNA were used as the negative controls. A CCK-8 assay was performed to evaluate whether MDHDH affects the viability of glioma cells. As shown in Fig. 2A, cell growth was significantly inhibited in both U87 and U251 cells when MDHDH was overexpressed. To further clarify whether the reduction in cell viability was caused by impeded cell proliferation or apoptosis, an EdU cell proliferation assay was applied. As depicted in Fig. 2B, the number of EdU-positive cells was significantly decreased (U87 NC vs. OE MDHDH: *P*=0.0098; U251 NC vs. OE MDHDH: *P*=0.002;) when MDHDH was overexpressed in both cell lines.

Transwell and wound healing assays were subsequently conducted to investigate whether MDHDH contributed to the migration and invasion of glioma cells. The results show that the migratory and invasive capabilities of U87 and U251 cells were remarkably suppressed by upregulation of MDHDH (Transwell migration: U87 NC vs. OE MDHDH: *P*<0.0001; U251 NC vs. OE MDHDH: *P*<0.0001; Transwell invasion: U87 NC vs. OE MDHDH: *P*<0.0001; U251 NC vs. OE MDHDH: *P*<0.0001; Wound healing: U87 NC vs. OE MDHDH: *P*=0.002; U251 NC vs. OE MDHDH: *P*=0.0083; Fig. 2C and D). Nevertheless, due to the low basal level of MDHDH expression, the GBM cells with MDHDH knockdown only had moderate effects on these malignant phenotypes (Supplementary Figure S3B-F), even though the efficiency of MDHDH knockdown was statistically significant (Supplementary Figure S3B). In addition, a nude mouse tumor-bearing model of glioma formation in situ was constructed. The results confirm that the tumor formation derived from MDHDH-overexpressing U87 cells was significantly suppressed (Total flux 7d NC vs. MDHDH:*P*=0.0031; 14d NC vs. MDHDH:*P*=0.0033), and overexpression of MDHDH prolonged the survival time of the experimental animals (NC vs. MDHDH: log-rank *P*=0.0018) (Fig. 2E). Notably, we obtained similar results in C57 mice using the MDHDH-overexpressing GL261 cell line in pilot experiments (Supplementary Figure S4A). Therefore, using NCBI-BLAST for MDHDH sequence alignment, we found a sequence similar to MDHDH on the X chromosome of C57 mice (Supplementary Figure S4B). This conserved sequence became the basis for our subsequent RNA truncation. Collectively, these experiments suggest that MDHDH inhibited the malignant phenotypes of GBM cells.

### Identification of MDH2 and PSMA1 as the binding proteins for MDHDH

**Fig. 3:**
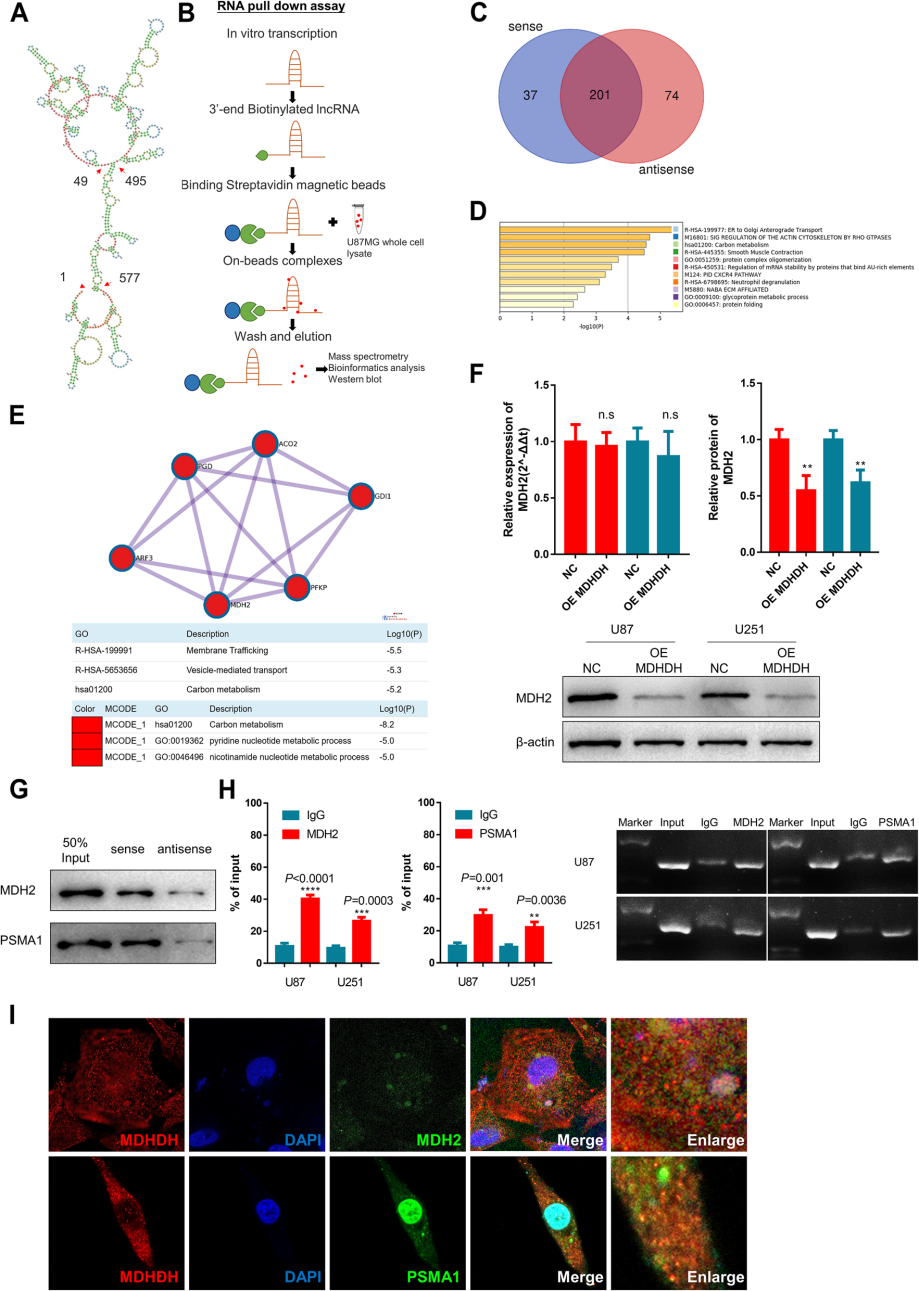
Identification of MDH2 and PSMA1 as the binding proteins for MDHDH. **A** Predicted secondary structure of MDHDH generated by the NONCODE built-in online tool. Some of the break-points of fragments used in pulldown were marked (arrowheads) with nucleotide numbers: 1–293, 294–564, 565–749, 1-49/495-577, 50-494 and 578-749. Each nucleotide is color-coded. **B** Schematic illustration of the RNA pulldown assay followed by mass spectrometry analysis. **C** Venn diagram illustrating the eluted proteins of the MDHDH full-length sense RNA probe and antisense RNA probe detected by mass spectrometry analysis (sense: 238, antisense: 275, sense-only: 37). **D** Metascape enrichment analysis and PPI network for the list of proteins that specifically bind to the full-length sense RNA probe (upper). Pathway enrichment results (bottom). **E** Molecular complex detection (MCODE) was applied to explore the significant modules in the PPI network. MCODE and GO results and description (bottom). **F** Western blot analysis indicating that MDH2 was regulated by MDHDH. qRT–PCR results indicate that the expression level of MDH2 was not affected by the overexpression of MDHDH. The regulation of MDH2 by MDHDH is a posttranscriptional regulation. **G** Western blot analysis showing the interaction between MDHDH, MDH2 and PSMA1. MDHDH antisense eluted protein was used as a negative control. **H**, RIP assays were performed with U87 and U251 cell extracts using anti-MDH2, anti-PSMA1 or mouse IgG. IgG served as the negative control. RNAs enriched in anti-MDH2, anti-PSMA1 and IgG pulldowns were determined relative to the input control. Agarose gel electrophoresis of RIP-PCR products (bottom). **I** FISH probe of MDHDH (red) costained with MDH2/PSMA1 (green) fluorescence. Fluorescence assessment of MDHDH colocalized (yellow) with MDH2/PSMA1

To investigate the molecular mechanism by which MDHDH exerted its effects on GBM cells, an RNA pulldown assay followed by mass spectrometry (MS) was performed to identify other MDHDH-associated proteins that might be involved in MDHDH-related biological processes (Fig. 3A and B). The MS results show that 238 and 275 proteins interact with the MDHDH sense and antisense RNA probes, respectively. After comparison, we found that a total of 37 proteins specifically bound to MDHDH (Fig. 3C). Based on the protein list (238 proteins) of the RNA pulldown, we used the Metascape online database to construct a protein–protein interaction (PPI) network and performed gene/pathway enrichment analysis (Fig. 3D and Supplementary Figure S4C-E) using MCODE (Fig. 3E). The results show that the main function intersection was carbon metabolism (Supplementary Table 13). Moreover, we assessed whether MDHDH influenced the expression level of MDH2. The results show that overexpression of MDHDH significantly reduced MDH2 protein levels (Fig. 3F, bottom). However, the transcriptional level of MDH2 was unaffected, suggesting that MDHDH might regulate MDH2 at the posttranscriptional level in U87 and U251 cells.

There are two main mechanisms of intracellular protein degradation: the ubiquitin–proteasome system (UPS) and the autophagy–lysosome pathway (ALP). Among the hub genes from MCODE and the genes with the highest frequency of pathway enrichment, we noticed that the composition of the proteasome subunit PSMA1 was in the list of enriched genes that interacted with the MDHDH sense chain. According to the above findings, we proposed a hypothetical model in which the posttranscriptional degradation of MDH2 might be achieved through the ubiquitin–proteasome system. Then, we verified that the sense MDHDH, but not the antisense, specifically bound to MDH2 and PSMA1. We next silver stained the proteins derived from the RNA pulldown assay and analyzed the differential bands using MS for double verification. The results of silver staining and Western blotting both indicate that MDH2 and PSMA1 interacted with MDHDH (Fig. 3G, Supplementary Figure S4F and Supplementary Table 14). The RIP assay followed by agarose gel electrophoresis of the PCR products further confirmed the above results (Fig. 3H). Fluorescent costaining results using a FISH probe of MDHDH with MDH2/PSMA1 also confirmed the presence of MDHDH bound to MDH2/PSMA1 (Fig. 3I). Mitochondrial isolation and subsequent PCR experiments also verified the enrichment of MDHDH in mitochondria (Supplementary Figure S4G). In short, the above results show that MDHDH bound to MDH2 and PSMA1. Moreover, upregulation of MDHDH expression led to a decrease in the protein level of MDH2.

### MDHDH promoted the interaction of MDH2 and PSMA1

**Fig. 4:**
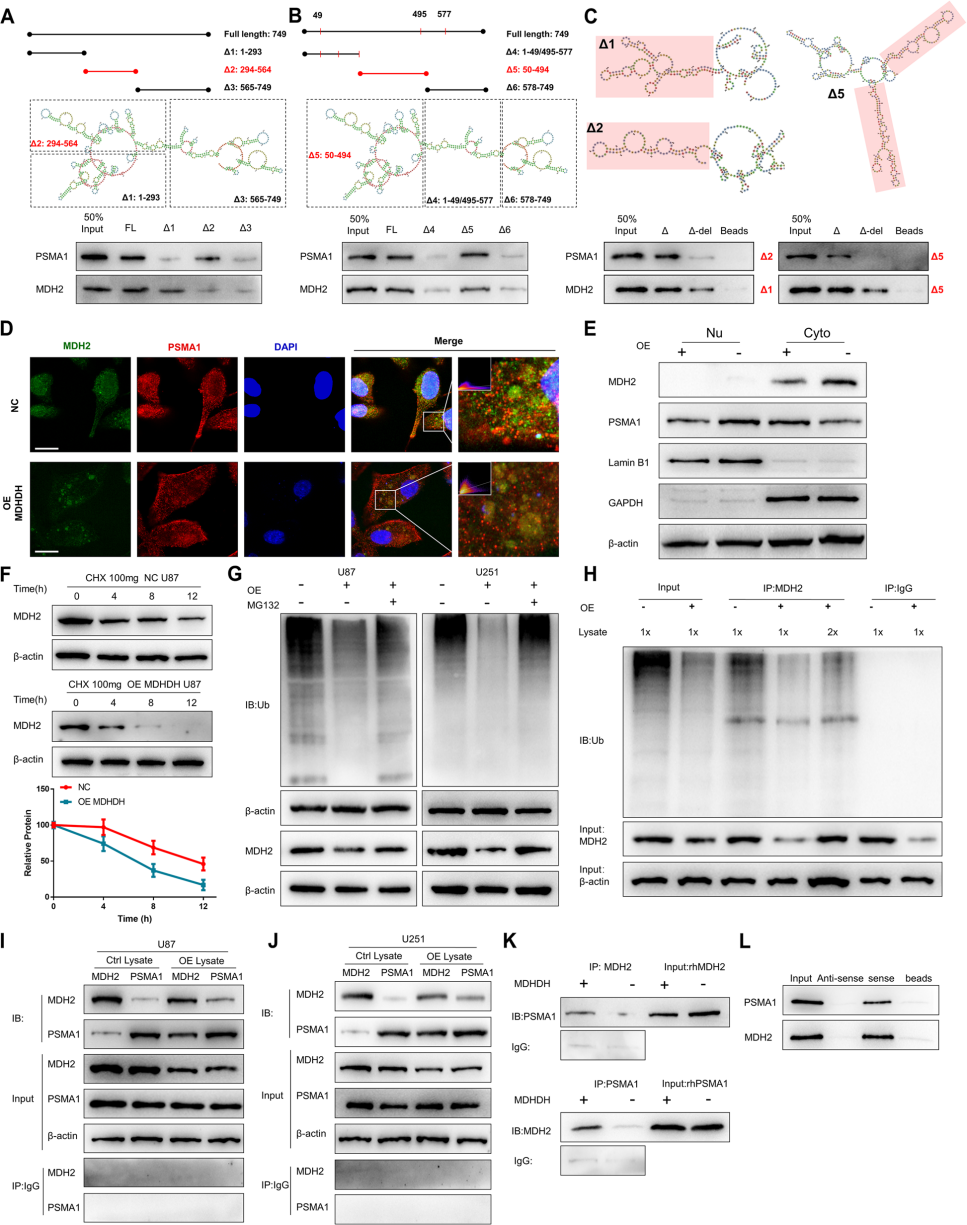
MDHDH binds MDH2 and PSMA1, and promotes their colocalization and MDH2 degradation by promoting the binding of ubiquitinated MDH2 and PSMA1. (A and B), Schematic diagram of the truncated biotin-labeled MDHDH RNA probe (upper). Western blot analysis showing the interaction between truncated MDHDH and MDH2 or PSMA1 (bottom). MDH2 mainly interacted with Δ1, PSMA1 mainly interacted with Δ2, and both interacted with the RNA main stem–loop structure (Δ5). (C), Secondary structure of RNA truncation probes (Δ1, Δ2, Δ5). The red frame section shows the key stem–loop structure of each truncated probe. Construction of truncated probes with deletion mutations (Δ-del). RNA pulldown experiments showed that the binding of three RNA probes (Δ1-del, Δ2-del, Δ5-del) to MDH2 and/or PSMA1 was diminished after deletion of the stem–loop structure. (D), Fluorescence assessment of MDH2 (green) and PSMA1 (red) enhanced colocalization (yellow) by MDHDH in U87 cells (scale bar = 10 μm, correlation scatter plot shown in the same panel). (E), Western blot of MDH2/PSMA1 using cytoplasmic and nuclear lysates isolated from control and MDHDH-overexpressing modified U87 cells to examine the effect of MDHDH on the subcellular location of MDH2 and PSMA1. (F), Western blot to detect MDH2 after 0, 4, 8 and 12 hours of cycloheximide (CHX, 100 mg/mL) treatment in the control and MDHDH-overexpressing U87 cells. Decay curve of MDH2 protein in the control and MDHDH-overexpressing U87 cells based on semiquantitative analysis of bands in the left panel. (G), U87 and U251 cells expressing either MDHDH or the control vector were cultured in the presence or absence of MG132 (20 μM) for 6 h. The cell lysates were analyzed by Western blotting with an anti-ubiquitin antibody. (H), Lysates from U87 cells transfected for MDHDH overexpression or with the control vector were subjected to immunoprecipitation with anti-MDH2 antibody or mouse IgG followed by immunoblotting analysis with anti-ubiquitin antibody. The MDH2 expression level of the overexpression group was approximately 50% of that of the control group, and the double eluate of the overexpression group was added to reflect the ubiquitination level of MDH2 in the overexpression group. (I and J), Western blot of MDH2/PSMA1 Co-IPs using cell lysates isolated from the control and MDHDH-overexpressing modified U87 and U251 cells to examine the effect of MDHDH on the interaction of MDH2 and PSMA1. (K and L), Co-IP and RNA pulldown experiments in a cell-free system. The cell-free system utilized recombinant proteins instead of cell lysate, and the binding of recombinant MDH2 and recombinant PSMA1 was enhanced by MDHDH

To further explore the mechanism by which MDHDH regulates MDH2 or PSMA1, the public bioinformatics database catRAPID [40, 41] was utilized, which predicted that the binding sites of MDH2 and PSMA1 with MDHDH (Supplementary Figure S5) were different. Therefore, we constructed MDHDH truncation mutants in two ways according to the conservative analysis and the predicted secondary structure of MDHDH (Supplementary Figure S4B). The first three mutants were based on the conserved sequence of MDHDH (Δ1: 1-293; Δ2: 294-564; Δ3: 565-749), and the other three mutants were based on the maximum retention of the stem–loop structure (Δ4: 1-49/495-577; Δ5: 50-494; Δ6: 578-749). The results show that PSMA1 interacted with Δ2 and Δ5, and MDH2 bound to Δ1 and Δ5 (Fig. 4A and B). Since both proteins bound to the Δ5 region of MDHDH, it was highly possible that MDH2 and PSMA1 interacted with both sides of the same stem–loop structure. We next designed truncation probes for deletion mutations (Δ-del, Δ1-del:1-47/181-293; Δ2-del: 390-564; Δ5-del: 181-293/390-494) based on key stem–loop structures. The two stem–loop structures (48-180 and 294-389) were further clarified to bind to MDH2 and PSMA1 (Fig. 4C), respectively. For the details of truncated RNA probe sequences, truncated RNA probe sequences after deletion mutations, and the secondary structure of the above probes, please refer to the supplemental figure (Supplementary Figure S6). In addition, the immunofluorescence results indicate that upregulation of MDHDH promoted the colocalization of MDH2 and PSMA1 in glioma cells, further suggesting that MDHDH plays a role in the interaction between MDH2 and PSMA1 (Fig. 4D).

### MDHDH promoted the degradation of MDH2 by inducing the interaction between ubiquitinated MDH2 and PSMA1

PSMA1 stands for proteasome subunit alpha type-1; therefore, it is self-evidently involved in the UPS. Considering that MDHDH decreased the protein instead of RNA expression level of MDH2, we proposed that MDHDH might take part in accelerating the degradation of MDH2 by enhancing the interaction of ubiquitinated MDH2 and proteasome subunits PSMA1. To prove this hypothesis, a nuclear-cytosol extraction assay was performed. The results show that even though PSMA1 existed mainly in the nuclear extracts of control cells, the upregulation of MDHDH could induce PSMA1 translocation to the cytoplasmic portion where MDH2 was localized (Fig. 4E). When the protein synthesis inhibitor cycloheximide (CHX) was applied, the U87 cells overexpressing MDHDH exhibited a shorter MDH2 half-life than the negative control cells (Fig. 4F). On the other hand, treatment with the proteasome inhibitor MG-132 significantly reversed the reduction in the ubiquitinated smear of total proteins induced by MDHDH overexpression (Fig. 4G and Supplementary Figure S7A), suggesting that MDHDH was involved in the degradation of ubiquitinated proteins.

To clarify the specific role of MDHDH in the degradation of MDH2 (resulting from either the enhanced ubiquitination of MDH2 itself or the promotion of MDH2 binding to the proteasome complex), we performed a ubiquitination immunoblot experiment upon MDH2 immunoprecipitation [42] (Fig. 4H). Considering that the overexpression of MDHDH caused an approximate decrease of 50% in the amount of MDH2, we added an extra lane with twice the loading volume so that the initial level of ubiquitinated MDH2 was equal between each group. The results show that enhanced expression of MDHDH did not affect the ubiquitination level of MDH2 (Fig. 4H and Supplementary Figure S7B). Nevertheless, it promoted the binding capacity of MDH2 to PSMA1, evidently consistent with the previous results (Fig. 4I and J). Meanwhile, to verify that MDHDH and PSMA1/MDH2 directly interact, we performed validation using a cell-free system. The proteins in the cell-free system were obtained from commercial recombinant proteins to exclude the effect of other components in the cell lysate. The results of the cell-free system are consistent with the results of previous experiments (Fig. 4K and L). In summary, the mechanism of MDHDH in the regulation of MDH2 degradation might be achieved by increasing the interaction of ubiquitinated MDH2 with PSMA1 rather than enhancing the ubiquitination of MDH2.

### MDHDH regulated the bioenergetic supply of glioma cells and promoted cell autophagy and apoptosis via the AMPK/mTOR pathway

As a key enzyme of MAS, MDH2 is considered the major NADH shuttle in the brain [10]. NADH, the reduced form of NAD+, plays a significant role in redox reactions in energy metabolism [43]. The presence of MDH2 through MAS is essential to maintain the necessary NAD+/NADH ratio required for glycolysis. Under this circumstance, we hypothesized that the degradation of MDH2 induced by MDHDH might affect the bioenergetic supply of the GBM cell lines, thereby regulating their metabolic reprogramming.

Cancer cells have unique NAD+ metabolic pathways dependent on tissue specificity and genotype. We first examined the NAD+ levels and NAD+/NADH ratios in the cytosol of glioma cells. The results show that MDHDH significantly decreased NAD+ levels and NAD+/NADH ratios in both U87 and U251 cells (Supplementary Figure S8A-C). To determine the effects of MDHDH on anaerobic glycolysis, extracellular pyruvate and lactic acid assays were conducted. The results show that overexpression of MDHDH significantly decreased the extracellular pyruvate and lactate levels of GBM cell lines (Supplementary Figure S8D and E).

To further elucidate whether MDHDH was involved in the metabolic reprogramming of glioma cells, XFe24 analysis (Seahorse) was performed. The results show a lower consumption rate of oxygen (OCAR) and decreased extracellular acidification (ECAR) (Supplementary Figure S8F) when MDHDH was upregulated. Mitochondrial membrane potential (ΔΨm) was subsequently examined by incubating the JC-1 (5,5',6,6'-tetrachloro-1,1',3,3'-tetraethylbenzimi-dazolylcarbocyanine iodide) probe [44] with different cells (Supplementary Figure S8G). CCCP (carbonyl cyanide 3-chlorophenylhydrazone) treatment was used as a positive control. Compared with the NC group, upregulation of MDHDH significantly decreased the mitochondrial potentials, suggesting that MDHDH also affected the function of mitochondria in glioma cells.

The above results evidently suggest that MDHDH had significant inhibitory effects on the anaerobic glycolysis and energy production of the glioma cells. Therefore, we evaluated the signaling pathways related to the energy metabolism of the cells.

In eukaryotic cells, AMP-activated protein kinase (AMPK) is a key energy sensor that regulates cellular metabolism to maintain energy homeostasis. The downstream mTOR pathway regulates autophagy in response to the energy response of AMPK. Therefore, we investigated the changes in the AMPK and mTOR pathways by examining the expression levels of proteins involved in MDHDH-upregulated (OE MDHDH) U87 cells, negative control U87 cells and U87 cells with glucose-free medium starved for 4 h (as a positive control). The results show that the expression of p-AMPK and p-ACC ((phospho-)acetyl-coA carboxylase) in the OE MDHDH group was significantly increased, while the level of downstream p-mTOR was markedly decreased (Supplementary Figure S8H).

Furthermore, we used the mCherry-GFP-LC3B fusion protein to measure the changes in autophagic flux. The change in fluorescence signal shows the formation of autophagosomes and the process of lysosome binding (yellow- no autophagy; green puncta- autophagosome formation; red puncta- autophagy lysosome formation). Fluorescence imaging showed that a large number of green puncta were formed in the MDHDH-overexpressing cells compared with the control cells. However, the red fluorescent signal did not aggregate (Supplementary Figure S8I), suggesting that MDHDH is involved in the regulation of autophagy in glioma cells. Correspondingly, as an important biomarker of autophagy, LC3B-I/II was also upregulated accordingly, indicating an increased level of autophagy [45]. Compared with control cells, the expression level of LC3B-II also significantly increased (Supplementary Figure S8H). The metabolic changes involved in MDHDH may be associated with altered transcriptional levels of HIF1A. Similar to another study [17], the reduction or loss of function of MDH2 resulted in a decrease in the transcriptional levels of HIF1A, which in turn affected the transcription of other key enzymes of glycolysis (Supplementary Figure S8J). This may be an additional factor contributing to the regulatory mechanism of MDHDH.

### PSMA1 was involved in the regulation of glioma by MDHDH

**Fig. 5:**
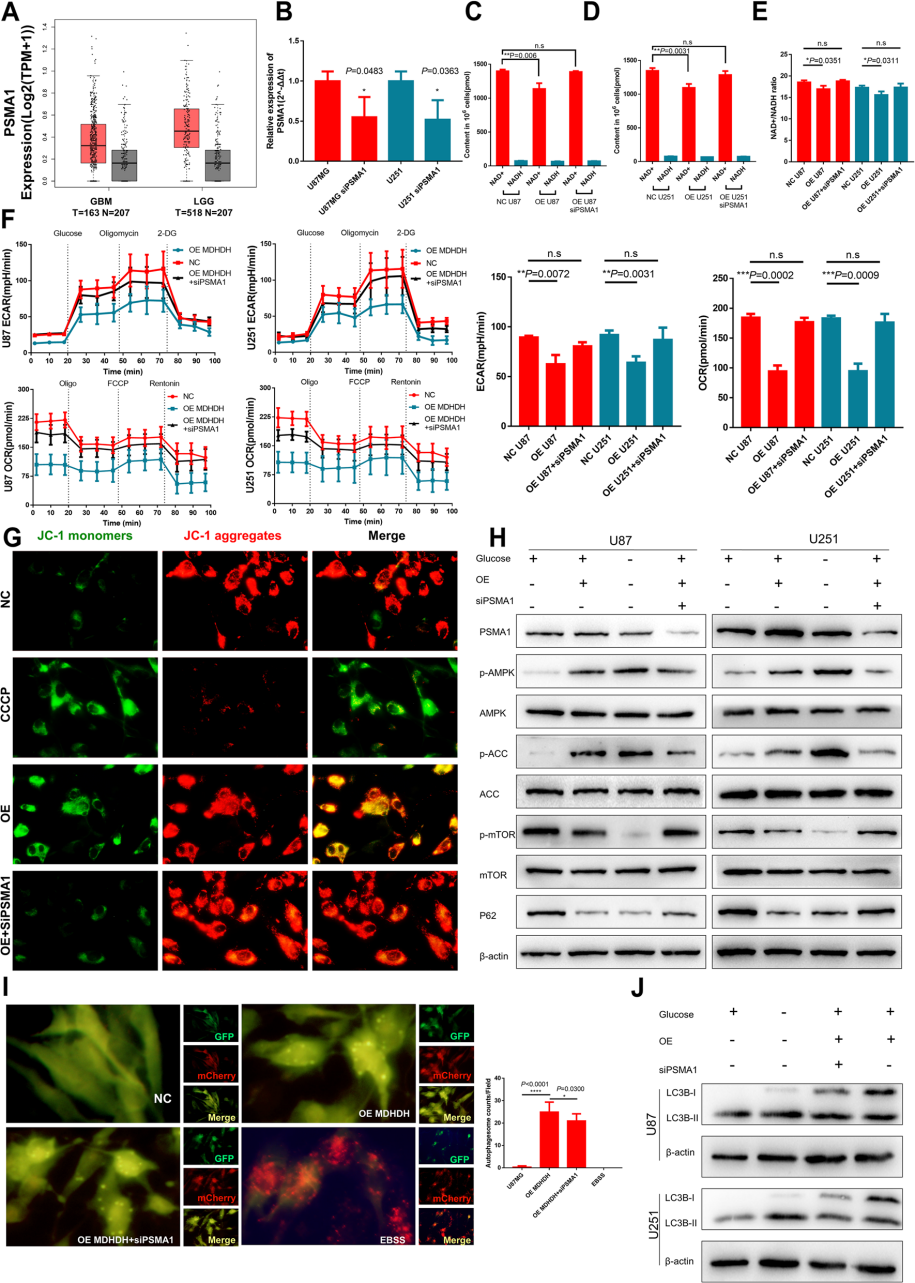
PSMA1 was required for MDHDH regulation of GBM cells and the AMPK/mTOR pathway. **A**, Graphic representation of the relative PSMA1 expression level (TPM) in different tissues (GBM vs. GTEx, LGG vs. GTEx). **B** U87 and U251 cells were transfected with PSMA1 siRNA and compared with the basal PSMA1 expression level of the control groups. The knockdown efficiency was analyzed with qRT–PCR, and β-actin served as the internal control. **C**-**E** NAD+ and NAD+/NADH ratio in MDHDH-, MDHDH+siPSMA1- or negative control vector-transfected U87 and U251 cells (Student’s t test; column means of triplicate assays). **F** OCR and ECAR in MDHDH-, MDHDH+siPSMA1- or negative control vector-transfected U87 and U251 cells. The OCR and ECAR were measured using a Seahorse analyzer. **G** The mitochondrial transmembrane potential was tested using JC-1 fluorescent probes of U87 cells transfected with MDHDH, MDHDH+siPSMA1 or negative control vector. CCCP-treated U87 cells were used as a positive control (scale bar = 50 μm; column means of triplicate assays). **H** PSMA1 was required for the MDHDH-regulated AMPK/mTOR pathway and promoted glioma autophagy. The lysates of GBM cells cultured with glucose-free DMEM for 2 h were used as the positive control group. **I** Representative images of U87 cells transfected with Ad-mCherry-GFP-LC3B adenovirus after transfection with the MDHDH overexpression vector (OE MDHDH group), MDHDH overexpression vector + PSMA1 siRNA (OE MDHDH+siPSMA1 group) or control vector (NC group). U87 cells cultured in EBSS medium for 24 hours were used as a positive control (scale bar = 10 μm). (J), Western blot of LC3B-I/LC3B-II in MDHDH-, MDHDH+siPSMA1- and control modified U87 and U251 cells

To investigate the role of PSMA1 in the functions of MDHDH, we performed rescue studies in U87 and U251 cells with PSMA1 knockdown. According to the GBM and LGG datasets in TCGA, the expression level of PSMA1 in tumors was higher than that in normal brain tissues, although the difference was not statistically significant (Fig. 5A). We constructed a cellular model of PSMA1 downregulation using siPSMA1 and repeated the experiments. As a result, we found consistent with the results in the previous section: MDHDH overexpression could effectively reduce NAD+ levels and alter the NAD+/NADH ratio (NAD+ U87 NC vs. OE: P=0.006, U251 NC vs. OE: P=0.0031; NAD+/NADH ratio U87 NC vs. OE: P=0.0351, U251 NC vs. OE: P=0.0311), but these changes could be rescued by PSMA1 downregulation (NAD+ and NAD+/NADH ratio U87/U251: NC vs. OE+siPSMA1: no significance). When PSMA1 was downregulated in U87 and U251 cells the changes of OCR, ECAR caused by overexpression of MDHDH (Fig. 5C-F), were attenuated. Overexpression of MDHDH significantly reduced the ECAR and OCR levels in glioma cells, and the ECAR and OCR levels in the cell model after overexpression of MDHDH and knockdown of PSMA1 were close to those in the control group (ECAR: U87 NC vs. OE: *P*= 0,0072 NC vs. OE+siPSMA1: no significance U251 NC vs. OE: *P*= 0.0031 NC vs. OE+siPSMA1: no significance OCR U87 NC vs. OE: *P*= 0.0002 NC vs. OE+siPSMA1: no significance U251 NC vs. OE: *P*= 0.0009 NC vs. OE+siPSMA1: no significance) (Fig. 5F). The results of mitochondrial membrane potential showed that the alterations brought about by MDHDH overexpression (JC-1 monomer, green fluorescence signal intensity) were rescued after PSMA1 downregulation (Fig. 5G). In addition, we repeatedly used the mCherry-GFP-LC3B fusion protein to measure the changes in autophagic flux, MDHDH overexpression-induced increase in autophagosomes can be reversed by PSMA1 knockdown (Fig. 5I). Meanwhile, autophagy level and the expression levels of key biomarkers involved in AMPK/mTOR pathway were restored as well (Fig. 5H and J).

**Fig. 6:**
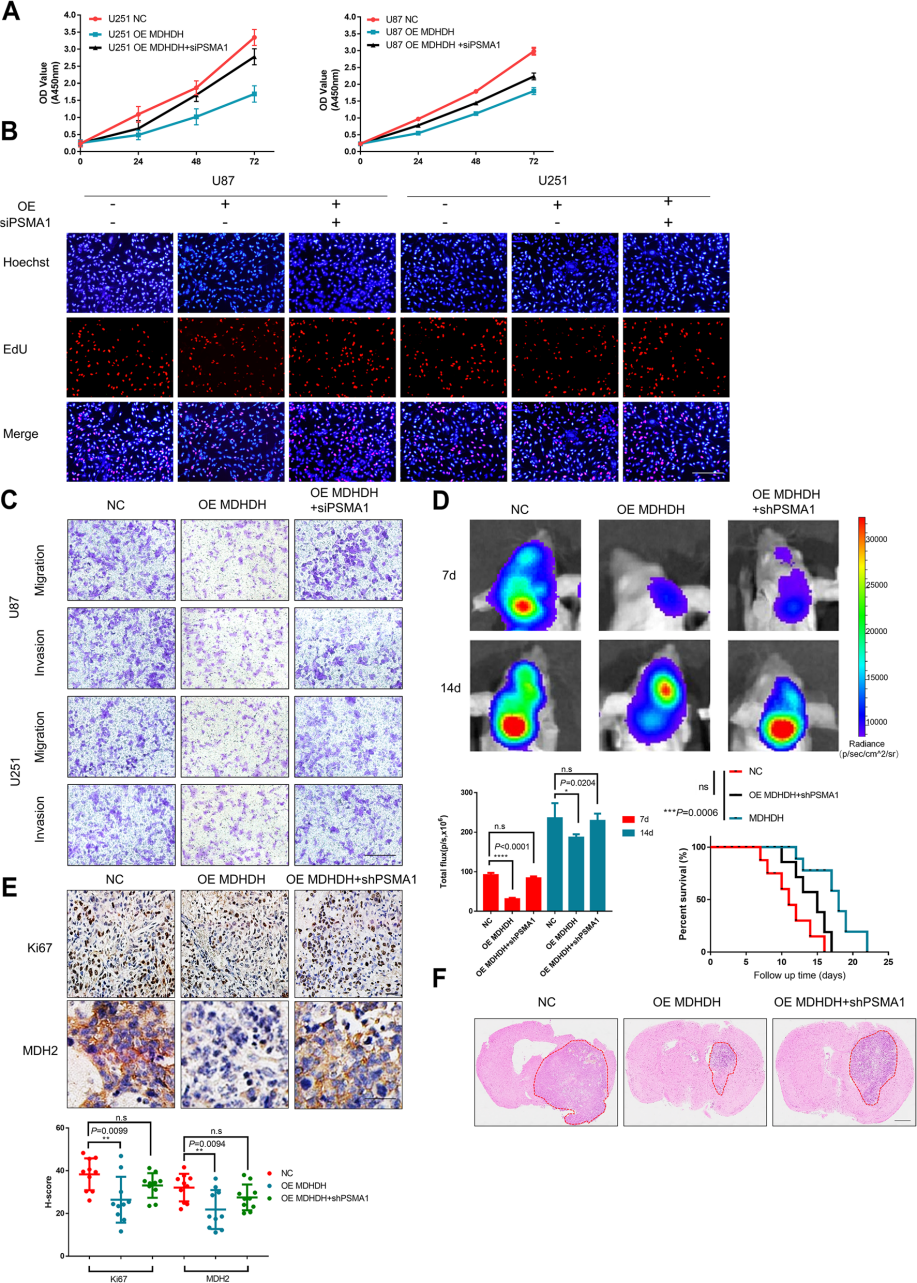
MDHDH suppresses the growth of GBM in vitro and in vivo via PSMA1. **A** U87 and U251 cells were transfected with the indicated plasmid constructs, and GBM cell line viability was examined by CCK-8 assay (left panel: U87 cell line, right panel: U251 cell line). **B** U87 and U251 cell (NC, OE MDHDH and OE MDHDH+siPSMA1) proliferation determined by EdU staining assay. **C** Migration and invasion of the transfected U87 and U251 cell lines (NC, OE MDHDH and OE MDHDH+siPSMA1) determined by transwell assay. **D** In vivo luminescent imaging of in situ tumor-bearing nude mice at the indicated time points. The total flux of the nude mice was counted 7 days and 14 days after intracranial implantation of luciferase-tagged U87 cells transfected with MDHDH, MDHDH+shPSMA1 or control plasmids by IVIS spectrum. The survival curves of the nude mice with xenografted tumors intracranially were recorded for 15 days. **E** Immunohistochemistry of Ki67 and MDH2 comparing NC, OE MDHDH or OE MDHDH+shPSMA1 (upper). Ki67 and MDH2 H-scores of different xenograft tissues (bottom). **F** Representative micrographs of HE-stained sections of mouse brain tissues under low- (scale bar = 250 μm) and high-power (scale bar = 200 μm) magnification 15 days after intracranial implantation of U87 cells infected with a lentiviral vector expressing NC, OE MDHDH or OE MDHDH+shPSMA1

We further investigated whether PSMA1 influenced the MDHDH-mediated cellular behaviors of glioma cells. The results showed that downregulation of PSMA1 alleviated the inhibitory effects of MDHDH on cell proliferation, invasion and migration mediated by MDHDH overexpression (Fig. 6A-C). Regarding the intracranial tumor xenografts in nude mouse models derived from U87 cells, PSMA1 silencing significantly abolished the suppressive role of MDHDH in the tumor xenograft growth level (Total flux: 7d NC vs. OE: *P*<0.0001, NC vs. OE+shPSMA1: no significance; 14d NC vs. OE: *P*=0.0204, NC vs. OE+shPSMA1: no significance; Fig. 6D and F). The overall survival of nude mice was prolonged correspondingly in comparison to mice bearing tumor xenografts upregulated with MDHDH and this could be reversed by PSMA1 knockdown (NC vs. MDHDH: Log-rank *P*=0.0006, NC vs. OE MDHDH+shPSMA1: no significance Fig. 6D, bottom).

We additionally tested the expression of Ki67 and MDH2 in xenografts from each group. The immunohistochemical images and calculated H-score indicate that overexpression of MDHDH significantly reduced the expression levels of Ki67 and MDH2 in xenografts, which could be effectively reversed by simultaneous knockdown of PSMA1 in these cells (Ki67 H-score: NC vs. OE MDHDH: *P*=0.0099; NC vs. OE MDHDH+shPSMA1:no significance; MDH2 H-score: NC vs. OE MDHDH: *P*=0.0094; NC vs. OE MDHDH+shPSMA1:no significance; Fig. 6E). Next, we further investigated the role of PSMA1 in the regulation of the cellular behaviors of glioma cells. We propose that PSMA1 knockdown alone leads to increased protein levels of MDH2, thereby exerting cancer-promoting functions. Knockdown of PSMA1 led to enhanced cell activity (Supplementary Figure S9A) and increased proliferation, invasion and migration (Supplementary Figure S9B-D), which were consistent with MDH2 overexpression. The Western blot results confirm that PSMA1 knockdown resulted in increased MDH2 protein levels (Supplementary Figure 9E). In addition, knockdown of PSMA1 and overexpression of MDHDH reversed MDHDH-mediated MDH2 degradation (Supplementary Figure S9E). Notably, although there was no significant difference in PSMA1 in tumor vs. normal brain tissue, we found a better prognosis in the low-PSMA1 expression group when comparing overall survival (OS) and disease-free survival (DFS) according to PSMA1 grouping (Supplementary Figure S9F). Metabolism-related experiments (lactate, pyruvate, ECAR/OCR) also yielded the same conclusion, with all glioma cell lines with PSMA1 knockdown having more active metabolic functions (Supplementary Figure S9G-O).

Glioma stem cells (GSCs) are more representative compared to glioma cell lines, we complement the studies related to glioma stem cells. We obtained glioma stem cells (Supplementary Figure S10A) using specific media (DMEM/F12, with 2% B27, 25ng/mL bFGF, 25ng/mL EGF and 1% penicillin/streptomycin) and verified the changes in mRNA levels (CD133 U251 vs. GSC_U251_: *P*=0.0005; SOX2 U251 vs. GSC_U251_ *P*=0.0034) and protein levels of their stem-biological markers (Supplementary Figure S10B). MDHDH reduced the protein level of MDH2 in glioma stem cells, and down-regulation of PSMA1 rescued the protein level of MDH2. In terms of metabolic functions, changes in NAD+ as well as NAD+/NADH ratios showed similar results as in U87 and U251, NAD+ and NAD+/NADH ratios in glioma stem cells are effectively reduced by MDHDH, and again, downregulation of PSMA1 reversed this result (NAD+: GSC_U251_ vs. OE MDHDH GSC_U251_: *P*= 0.0026, GSC_U251_ vs. OE MDHDH + siPSMA1 GSC_U251_: no significance; NAD+/NADH ratio: GSC_U251_ vs. OE MDHDH GSC_U251_: *P*= 0.0061, GSC_U251_ vs. OE MDHDH + siPSMA1 GSC_U251_: no significance; Supplementary Figure S10E). Simultaneous ECAR and OCR data were able to yield the same conclusion (ECAR: GSC_U251_ vs. OE MDHDH GSC_U251_: *P*= 0.0067 GSC_U251_ vs. OE MDHDH + siPSMA1 GSC_U251_: no significance; OCR: GSC_U251_ vs. OE MDHDH GSC_U251_: *P*= 0.0030 GSC_U251_ vs. OE MDHDH + siPSMA1 GSC_U251_: no significance; Supplementary Figure S10F). Mechanistically we repeated RNA pull-down experiments including full-length as well as antisense full-length, segmented MDHDH with results consistent with glioma cell lines (Supplementary Figure S10G). Western blot results of biomarkers associated with the AMPK/mTOR pathway in glioma stem cells are consistent with those in glioma cell lines: pathway alterations by MDHDH could be reversed by PSMA1 downregulation (Supplementary Figure S10H).

These results evidently suggest that PSMA1 is involved in the effects of MDHDH in the regulation of GBM cells. PSMA1 could serve as a key effector in MDHDH-induced inhibition in glioma cells.

### The MDHDH locus was epigenetically silenced by PRC2/H3K27me3 in GBM

**Fig. 7:**
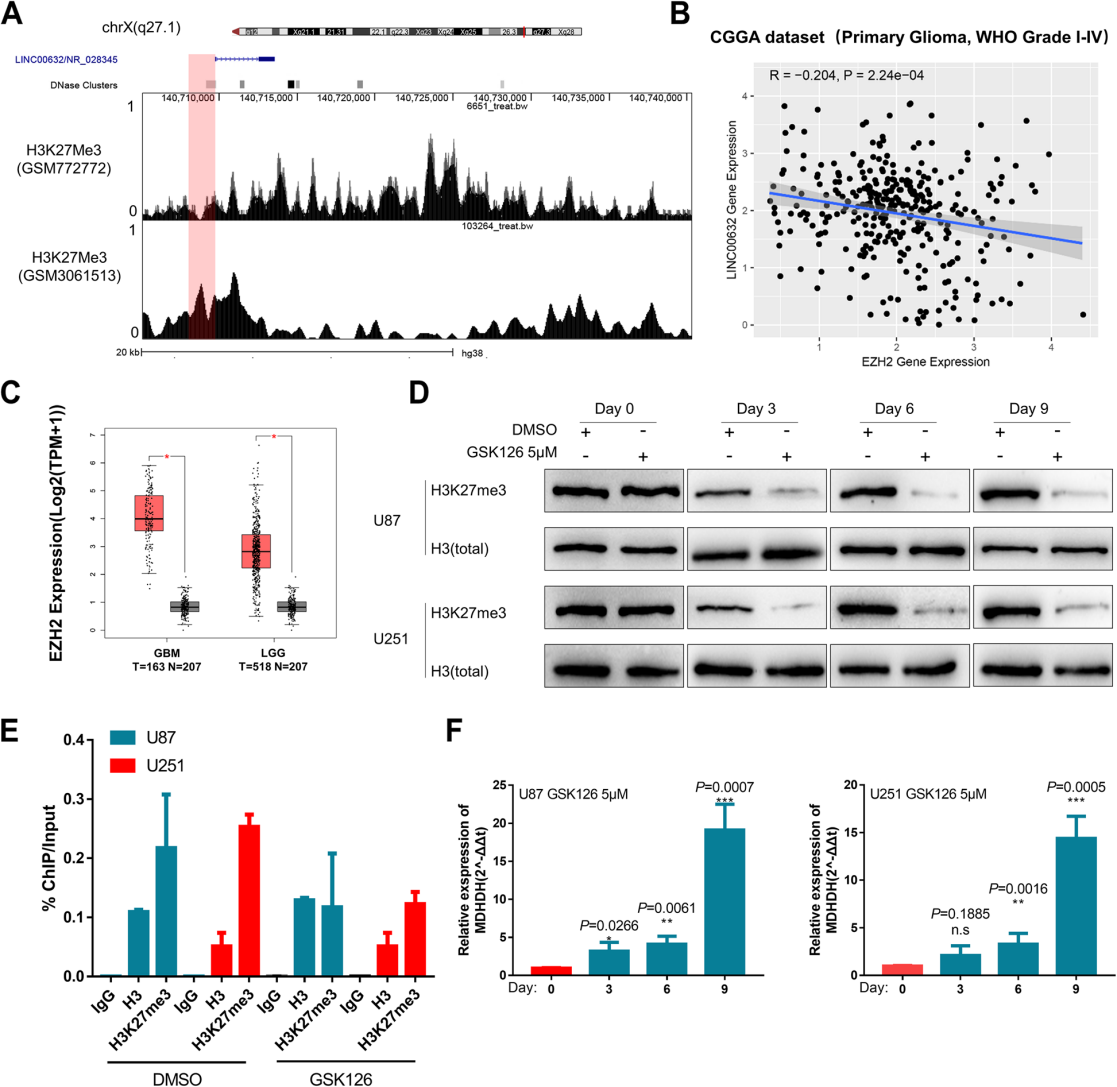
The MDHDH locus was epigenetically silenced by PRC2/H3K28me3 in glioblastoma multiforme. **A** ChIP-seq peak tracks of H3K27me3 from the Cistrome Data browser (T98G cell line, CistromeDB: 103264, normal brain tissue CistromeDB: 6651) in the MDHDH loci. **B** Pearson’s correlation of the CGGA primary glioma (WHO grade I-IV) expression profiles between EZH2 and LINC00632. **C** Graphic representation of the relative MDH2 expression level (TPM) in different tissues (GBM vs. GTEx, LGG vs. GTEx) (*, statistically significant). **D** Representative immunoblot of H3K27me3 and total histone H3 in EZH2 inhibitor-treated U87 and U251 cells. (E). Normalized abundance (%ChIP/Input, qPCR) of the TSS region from mouse IgG, H3, or H3K27me3 ChIP samples isolated from DMSO- or EZH2 inhibitor-treated (GSK126, 5 μM for 6 days) U87 and U251 cells. Mean ± SD of a representative experiment. **F** Relative expression of LINC00632 transcript variant 3 (MDHDH) in U87 and U251 cells treated with GSK126 (5 μM) for the indicated number of days. Data were normalized to the DMSO-treated control (first time point). Mean ± SD of a representative time course

We next sought to elucidate the molecular mechanisms that drive MDHDH silencing in high-grade gliomas. A previous study [46] reported that three transcript isoforms (ENST498732, ENST602535, ENST370535) of LINC00632 might be dependent on the chromatin state. In the publicly available chromatin immunoprecipitation sequencing (ChIP-seq) database (Cistrome Data browser) (Fig. 7A), H3K27 trimethylation (H3K27me3, a repressive histone mark) was abundant in the LINC00632 locus in the GBM cell line T98G. Therefore, we aimed to test whether H3K27me3 and EZH2 (a typical H3K27 methyltransferase) were responsible for the silencing of MDHDH in GBM.

The expression profile of a public glioma dataset (CGGA) shows that the expression of EZH2 had a negative correlation with the expression of LINC00632 (Fig. 7B). TCGA datasets also indicate that the expression level of EZH2 in gliomas was significantly higher than that in normal brain tissues (Fig. 7C). When GSK126, a specific EZH2 inhibitor, was used to treat glioma cells, the overall level of H3K27me3 in glioma cells was effectively decreased (Fig. 7D). By ChIP-PCR, we observed EZH2-dependent enrichment of H3K27me3 at the transcriptional start site (TSS) of MDHDH in GBM cell lines (Fig. 7E). Most strikingly, EZH2-dependent enrichment of H3K27me3 at the locus of MDHDH in GBM cell lines was also reduced after GSK126 was applied (Fig. 7E). A robust re-expression of MDHDH was induced thereafter (Fig. 7F). Collectively, these data demonstrate that EZH2-mediated H3K27me3 might serve as a major mechanism of MDHDH silencing in GBM cells.

## Discussion

Metabolic reprogramming is a hallmark of cancer cells. To promote growth, survival, proliferation, and long-term maintenance, cancer cells rewire/rewrite their metabolic modality. For gliomas, the metabolic and phenotypic changes brought by isocitrate dehydrogenase 1 and 2 (IDH1/2) mutations have become a consensus. A previous study confirmed that the presence of an IDH mutation might depend on depletion of the coenzyme NAD+ [47]; therefore, the process of NAD+ metabolism drew our attention. Increased anaerobic glycolysis in tumor cells is associated with alterations in NAD-related enzymes and glucose transporters in mitochondria. A high cytosolic NAD+/NADH ratio is therefore essential for maintaining this classic “Warburg effect” of cancers. MDH2 is one of the key cytoplasmic enzymes involved in MAS operation, which regulates the transition of NAD+/NADH by crossing mitochondria [15]. Previous studies have provided concrete evidence that the inhibition of MDH2 might provide a valuable platform for developing novel therapeutics that target cancer metabolism and tumor growth [16–18]. Based on the above, we focused on the key component of the MAS system, MDH2, to study its regulatory mechanism and potential effects on NAD+ metabolism in gliomas.

LncRNA transcriptome profiling is an effective approach to obtain a global view of cancers [48–50]. In the present study, we found that the downregulated lncRNA MDHDH interacted with MDH2 and was negatively correlated with the WHO grade classification of gliomas. MDHDH is a tumor suppressor associated with a better prognosis of patients with gliomas, and the overexpression of MDHDH resulted in significant inhibition of the proliferation, migration, and invasion of glioma cells.

Regarding the mechanism of MDHDH, quite different from other molecular scaffold studies, MDHDH did not directly affect the ubiquitination level of MDH2 but promoted the binding of ubiquitinated MDH2 to the proteasome subunit PSMA1. Therefore, our study might provide more evidence about the balance of protein stability of metabolic enzymes [51–53]. Moreover, we found that cytoplasmic MDH1 and other proteasome subunits (PSMA4/PSMB1) also potentially interacted with MDHDH (Supplementary Table 14 and Supplementary Figure 5), which suggests that MDHDH might act as a molecular chaperone involved in the UPS system. The phenotypic reversion caused by knockdown of PSMA1 may be associated with the blocked degradation and increased protein levels of MDH2, suggesting that UPS may also be involved in the regulatory aspects of metabolic function in glioma cells. The role of other proteasomal subunits needs to be further explored, and PSMA1, as one of the proteasomal subunits validated to bind to MDHDH, may be only partially involved in the function of MDHDH to exert MDH2 degradation.

We also investigated the alteration of NAD+ and the NAD+/NADH ratio in glioma cells, and further confirmed that the changes in metabolic status were induced by MDHDH-related MDH2 degradation. Considering the close relationship between NAD+ levels and the intracellular energy sensor AMPK, we further examined AMPK signaling. The results show that MDHDH promoted the activation of the AMPK/mTOR pathway, which in turn induced cell starvation and uncontrolled autophagy regulation. Based on the present results, our study of MDHDH expands the understanding of the mechanism of how lncRNAs regulate tumor metabolic reprogramming and autophagy, and provides a new perspective on the diagnosis and treatment of gliomas.

MDHDH is one of the transcriptional isoforms of LINC00632, which is located on chromosome X. To date, few studies have investigated the functions or molecular mechanisms of LINC00632 [46, 54–56]. It was generally confirmed that the sequence of ciRS-7 (circular RNA sponge for miR-7, also termed CDR1as, cerebellar degeneration-related protein 1 antisense RNA) was embedded in the LINC00632 locus [46, 57]. The expression of both LINC00632 and ciRS-7 was induced by an EZH2 inhibitor (EPZ-6438) [58]. In another study, several isoforms of LINC00632 were regulated by the epigenetic silencing of CDR1as and showed sex differences [46]. Inspired by the above studies, we further proved that PRC2/EZH2 mediated the epigenetic silencing of MDHDH in gliomas. Our study might provide another explanation as to why EZH2 inhibitors had potential antitumor effects. Effective epigenetic regulation would improve potential therapeutic value.

Interestingly, when we reviewed a study (GSE60666) [36] on the treatment of melanoma cell lines by (+)-JQ1 (thieno-triazolo-1,4-diazepine, a well-reported BRD4 (bromodomain-containing protein 4) inhibitor [59, 60]), we found that the cell line derived from male patients showed elimination of epigenetic silencing of LINC00632, while the data from female patients remained unchanged (Supplementary Figure S11A and B). In our study, we found that when the U87 cell line (with only one X chromosome activated) was treated with the EZH2 inhibitor GSK126, the autophagic level and mTOR pathway of the cells changed. Previous studies have reported that EZH2 inhibitors regulate autophagy [61–63]; however, the specific mechanism involved has not been elucidated in detail. This study provides an explanation for how GSK126 regulates autophagy. When the epigenetic inhibition of MDHDH in cells was relieved, the degradation of MDH2 protein and the subsequent ratio changes of NAD+/NADH would lead to autophagy. This suggests complicated regulatory relationships between the metabolic state, autophagy, and epigenetics (Supplementary Figure S11C). Moreover, we need to pay more attention to the sex differences between patients. Inactivation of the X chromosome in female patients would lead to more complicated tumor heterogeneity. For LINC00632 as well as MDHDH, the available evidence suggests a sex difference in LINC00632 in the TCGA-GBM dataset. LINC00632 expression was lower in female patients, which may be related to X-chromosome inactivation (XCI) (Supplementary Figure S11D). Data from PCR experiments on our clinical specimens showed a similar result. Differences were observed in three transcripts of LINC00632 (NR_028344.1, NR_104228.1 and NR_028345.2 (MDHDH)) from patients of different sexes, and all three exhibited lower expression levels in female patients (Supplementary Figure S11E). Notably, knockdown of MDHDH failed to result in phenotypic changes or metabolic alterations in cellular experiments (Supplementary Figures S3 and S12). However, based on TCGA-GBMLGG clinical subgroups (high-MDHDH group vs. low-MDHDH group), it appears that the impact of different expression levels on patient prognosis is still substantial due to the presence of heterogeneity (Fig. 1J and K). Notably, the same conclusion was obtained for our clinical cohort (Fig. 1L). Higher basal MDHDH expression levels may also result in insufficient sensitivity to proteasome inhibitor-based therapies, which may indirectly disrupt the downstream mechanism of MDHDH inhibition of tumor growth. Lower basal MDHDH expression levels imply that EZH2 inhibitor therapies may have more satisfactory results, but again, patients' gender and epigenetic suppression status need to be assessed. Therefore, assessment of the basal expression levels of MDHDH and sex factors may be necessary in the future application of GBM pharmacotherapy.

**Fig. 8:**
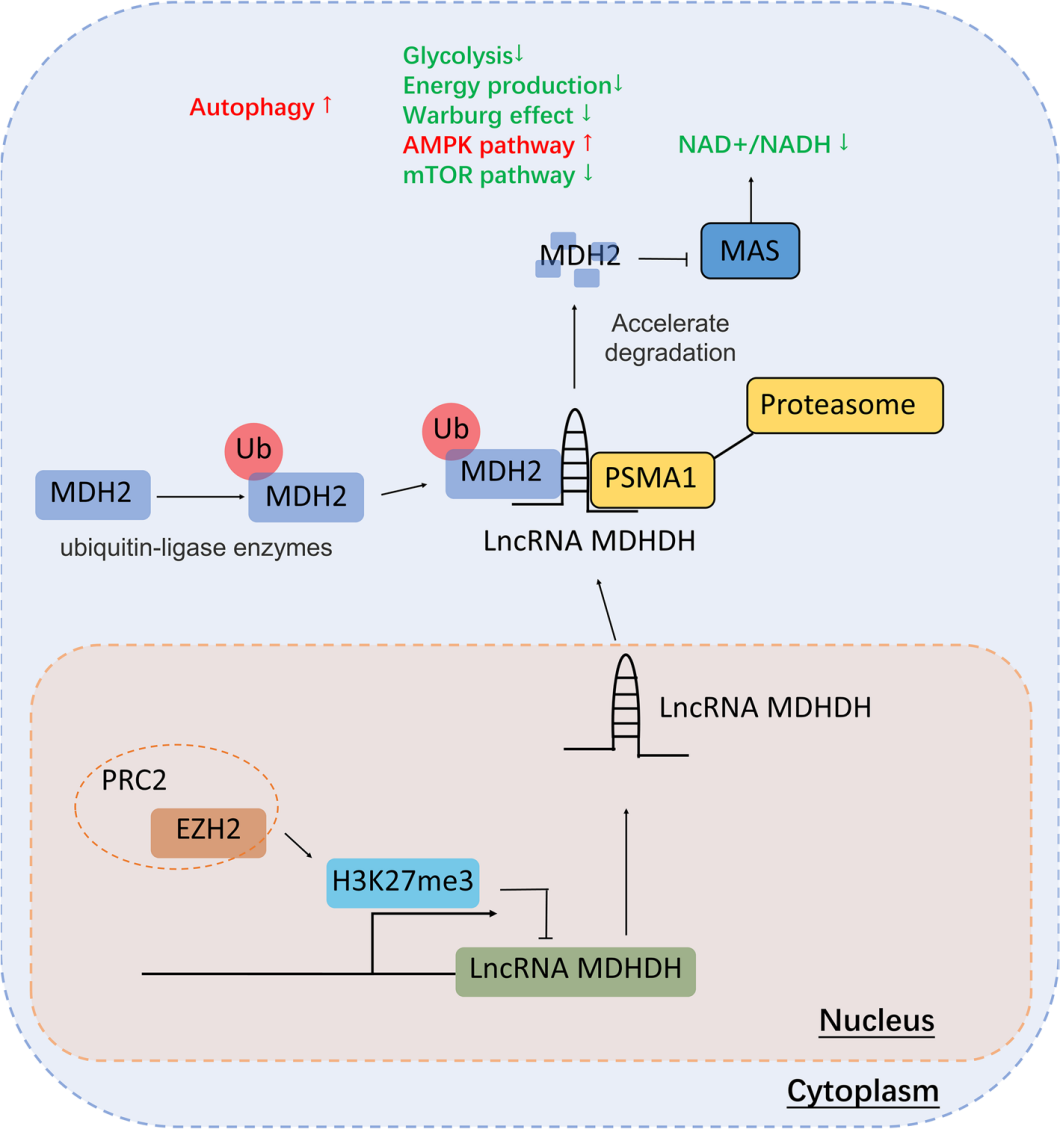
Graphical abstract illustrating MDHDH-induced GBM suppression. The reduced expression of MDHDH in GBM is attributed to EZH2-mediated epigenetic inhibition. MDHDH acts as a scaffold to promote binding of ubiquitin-modified MDH2 to PSMA1, triggering subsequent MDH2 degradation via the UPS. The degradation of MDH2 leads to the inefficacy of MAS, which is unable to maintain the essential NAD+/NADH ratio for substantial glycolysis. The abnormal metabolic state activates the AMPK/mTOR signaling pathway and induces autophagy to suppress GBM

In summary, our work not only uncovered the negative roles of MDHDH in GBM aggressiveness, metabolic reprogramming reversal and AMPK/mTOR pathway regulation but also implicated that MDHDH could act as a bridge to mediate autophagy-based and epigenetic-based therapies. These findings provide a systemic explanation for the changes in autophagy caused by EZH2 and BRD4 inhibitors. MDHDH has the potential to serve as a direct therapeutic target for GBM and an indicator for the evaluation of epigenetic therapies for GBM patients (Fig. 8).

## Conclusions

In conclusion, the present study broadened our understanding of the functions of lncRNAs in GBM. We demonstrated that the tumor suppressor MDHDH might act as a clinical biomarker and that the overexpression of MDHDH might be a novel synergistic strategy for enhancing metabolism-based, epigenetic-based, and autophagy regulation-based therapies, thereby fulfilling clinical benefits for glioblastoma multiforme patients.

## Supplementary Information

**Additional file 1.**

**Additional file 2.**

**Additional file 3.**

**Additional file 4.**

**Additional file 5.**

**Additional file 6.**

**Additional file 7.**

**Additional file 8.**

**Additional file 9.**

**Additional file 10.**

**Additional file 11.**

**Additional file 12.**

**Additional file 13.**

**Additional file 14.**

**Additional file 15.**

**Additional file 16.**

**Additional file 17.**

**Additional file 18.**

**Additional file 19.**

**Additional file 20.**

## Data Availability

The datasets used and/or analyzed during the current study are available from the corresponding author on reasonable request. Public databases or online tools were mentioned in this article. Please refer to Supplementary Table 15 for the raw data and results of ECAR/OCR and Fig. 4G/H for the grayscale analysis, pyruvate and lactate analyses, and EdU analysis.

## Abbreviations

(p-)ACC: (phospho-)acetyl-CoA carboxylase
(p-)AMPK: AMP-activated protein kinase
BCA: bicinchoninic acid
BRD4: Bromodomain-containing protein 4
CCCP: Carbonyl cyanide 3-chlorophenylhydrazone
CDR1as: CDR1 antisense RNA
CGGA: The Chinese Glioma Genome Atlas
CHX: cycloheximide
CISH: Chromogenic in situ hybridization
EBSS: Earle's balanced salt solution
ECAR: Extracellular acidification
EdU: 5-bromo-2'-deoxyuridine
EZH2: enhancer of zeste 2 polycomb repressive complex 2 subunit
FISH: Fluorescence in situ hybridization
GBM: Glioblastoma multiforme
GEPIA: gene expression profiling interactive analysis
GFP: Green fluorescent protein
GO: Gene ontology
GOT1/2: Glutamate oxaloacetate transaminase 1/2
GTEx: Genotype-Tissue Expression Project
H3K27me3: Histone H3 lysine trimethylation
HE: Hematoxylin-eosin
IDH1/2: Isocitrate dehydrogenase 1/2
JC-1: 5,5',6,6'-tetrachloro-1,1',3,3'-tetraethylbenzimi-dazolylcarbocyanine iodide
JQ-1: Thieno-triazolo-1,4-diazepine
LC3B1/2: Microtubule associated protein 1 light chain 3 beta
LGG: Low-grade glioma
MAS: Malate-aspartate shuttle
MCODE: Molecular complex detection
MDH1/2: Malate dehydrogenase 1/2
MDHDH: Malate dehydrogenase degradation helper
mTOR: mammalian target of rapamycin
NAD+: Nicotinamide adenine dinucleotide
NADH: Reduced nicotinamide adenine dinucleotide
NBT: Nitroblue tetrazolium
OCAR: Consumption rate of oxygen
P62: Sequestosome 1, SQSTM1
PPI: Protein–protein interaction
PRC2: Polycomb repressive complex 2
PSMA1/4: Proteasome 20S subunit alpha 1/4
PSMB1: Proteasome 20S subunit beta 1
ROC: Receiver operating characteristic
RTK: Receptor tyrosine kinase
TCGA: The Cancer Genome Atlas
UPS: Ubiquitin (Ub)-proteasome system
