# Supplementary material for: A novel lncRNA MDHDH suppresses glioblastoma multiforme by acting as a scaffold for MDH2 and PSMA1 to regulate NAD+ metabolism and autophagy
Source: J Exp Clin Cancer Res. 2022 Dec 17;41:349. doi: 10.1186/s13046-022-02543-7 (PMC9758949; doi:10.1186/s13046-022-02543-7)
Supplement: Supplementary file 2 — Additional file 2. [file 13046_2022_2543_MOESM2_ESM.docx]

**Supplementary Tables**

**Table 2 LncRNA MDHDH Reference Sequence information**

**Table 3 NCBI-ORFinder potential peptides alignment results**

**Table 4 Smart Silencer target sequences and FISH probe targets**

**Table 5 Primers list**

**Table 6 ROC baseline data**

**Table 7 LINC00632 expression in human glioma patients (TCGA-GBM and LGG) with different clinicopathological features. P values were determined by Chi-square and Wilcoxon tests.**

**Table 8 Correlation of LINC00632 expression in human glioma patients with different clinicopathological features. Odds Ratio and P value were determined by univariate logistic regression of LINC00632 expression.**

**Table 9 Univariate and multivariate Cox regression of LINC00632 expression for overall survival (OS) in glioma patients.**

**Table 10 Univariate and multivariate Cox regression of LINC00632 expression for disease specific survival (DSS) in glioma patients.**

**Table 11 Univariate and multivariate Cox regression of LINC00632 expression for progress free interval (PFI) in glioma patients.**

**Table 2 LncRNA MDHDH Reference Sequence information**

>NR_028345.2 Homo sapiens long intergenic non-protein coding RNA 632 (LINC00632), transcript variant 2, long non-coding RNA

AGTGCGACAGACAGCCTCAGAGCTGGTGGGGAGACAGCATGCCACTGGAAAAATTCGTAGACATGGAATTCCTGGATCAAATGGTATGAAACAATTTAAGGTTTCTTCCCCCCAACACCTATCACCTAATTTATTTATTATATGTGGTATAAACATTATTTTCCAGCCATTGTTTTCCTATTAATTTTTACCATTTATGGTGGTGATTTTTTGATATTTTACAGTTTTACATGTTAGATTTCCATATCTGACCATTTTAAGTGTTGTGATTTTTTTCTTTCCTTTGATTAGTGTATTCACCTGTATTTTTTTCTAGTTATTTAATTGCTTCATTTTTTACAATTAACTCCTTAATCCACCTAGAATTCATTTTGATATATGGTATACAGTAGGATTTAATTTTTTTTCCCCCTAATAGGTCACCGATTGTTTCAGCATCACTTATTGAATAATCCAACGCAACAACTGTTCTTCCATTAGATAGGGTGTAGCTTTCCACAGGTCGGCCATTTTCTCTTTACCCAAGATGGTTACATTTTGCAATGGTACTCCTACCTTCCTGCAGCTTCTGACAGCATACTAGAGCACCATGTTTGTGCTTGTGCGTTTCAAAAATCCTAGGCACATGCAGATTAGGGCAGTATTTTAAACAGGATTTTTTTTTTCATTTTAAATATGTTAAGTTTGTCACATTCTTGTTGCAAATTAAAATTAAGGAATCCAAATATATGAAACCACGGTTTCAGGTG

**Table 3 NCBI-ORFinder potential peptides alignment results**

RID: 814MUERN013 Job Title:Protein Sequence Program: Database: swissprot Non-redundant UniProtKB/SwissProt sequences

| Peptide product Result | |
| --- | --- |
| >lcl\|ORF1:142:237 unnamed protein product  MWYKHYFPAIVFLLIFTIYGGDFLIFYSFTC | Query #1: lcl\|ORF1:142:237 unnamed protein product Query ID: lcl\|Query_97459 Length: 31 No significant similarity found. |
| >lcl\|ORF2:676:732 unnamed protein product  MLSLSHSCCKLKLRNPNI | Query #2: lcl\|ORF2:676:732 unnamed protein product Query ID: lcl\|Query_97460 Length: 18 No significant similarity found. |
| >lcl\|ORF3:86:184 unnamed protein product  MKQFKVSSPQHLSPNLFIICGINIIFQPLFSY | Query #3: lcl\|ORF3:86:184 unnamed protein product Query ID: lcl\|Query_97461 Length: 32 No significant similarity found. |
| >lcl\|ORF4:527:583 unnamed protein product  MVTFCNGTPTFLQLLTAY | Query #4: lcl\|ORF4:527:583 unnamed protein product Query ID: lcl\|Query_97462 Length: 18 No significant similarity found. |
| >lcl\|ORF5:590:649 unnamed protein product  MFVLVRFKNPRHMQIRAVF | Query #5: lcl\|ORF5:590:649 unnamed protein product Query ID: lcl\|Query_97463 Length: 19 No significant similarity found. |
| >lcl\|ORF6:39:89 unnamed protein product  MPLEKFVDMEFLDQMV | Query #6: lcl\|ORF6:39:89 unnamed protein product Query ID: lcl\|Query_97464 Length: 16 No significant similarity found. |
| >lcl\|ORF7:668:633 unnamed protein product  MKKKILFKILP | Query #7: lcl\|ORF7:668:633 unnamed protein product Query ID: lcl\|Query_97465 Length: 11 No significant similarity found. |
| >lcl\|ORF8:194:156 unnamed protein product  MVKINRKTMAGK | Query #8: lcl\|ORF8:194:156 unnamed protein product Query ID: lcl\|Query_97466 Length: 12 No significant similarity found. |
| >lcl\|ORF9:370:317 unnamed protein product  MNSRWIKELIVKNEAIK | Query #9: lcl\|ORF9:370:317 unnamed protein product Query ID: lcl\|Query_97467 Length: 17 No significant similarity found. |
| >lcl\|ORF10:64:17 unnamed protein product  MSTNFSSGMLSPHQL | Query #10: lcl\|ORF10:64:17 unnamed protein product Query ID: lcl\|Query_97468 Length: 15 No significant similarity found. |
| >lcl\|ORF11:627:580 unnamed protein product  MCLGFLKRTSTNMVL | Query #11: lcl\|ORF11:627:580 unnamed protein product Query ID: lcl\|Query_97469 Length: 15 No significant similarity found. |
| >lcl\|ORF12:510:442 unnamed protein product  MADLWKATPYLMEEQLLRWIIQ | Query #12: lcl\|ORF12:510:442 unnamed protein product Query ID: lcl\|Query_97470 Length: 22 No significant similarity found. |
| >lcl\|ORF13:255:181 unnamed protein product  MVRYGNLTCKTVKYQKITTINGKN | Query #13: lcl\|ORF13:255:181 unnamed protein product Query ID: lcl\|Query_97471 Length: 24 No significant similarity found. |
| >lcl\|ORF14:156:1 unnamed protein product, partial  MFIPHIINKLGDRCWGEETLNCFIPFDPGIPCLRIFPVACCLPTSSEAVCRT | Query #14: lcl\|ORF14:156:1 unnamed protein product, partial Query ID: lcl\|Query_97472 Length: 52 No significant similarity found. |

**Table 4 Smart Silencer target sequences and FISH probe targets**

| Smart Silencer target sequences (5'-3') |
| --- |
| CTCCTTAATCCACCTAGAAT |
| TTTCCACAGGTCGGCCATTT |
| GACAGCATACTAGAGCACCA |
| GCAGATTAGGGCAGTATTT |
| CAACACCTATCACCTAATT |
| CGCAACAACTGTTCTTCCA |

Sequences Report S/N: PA201904100067

Control sequences information contact: Guangzhou Riobobio Co., LTD

**FISH probe and targets**

AGTGCGACAGACAGCCTCAGAGCTGGTGGGGAGACAGCATGCCACTGGAAAAATTCGTAGACATGGAATTCCTGGATCAAATGGTATGAAACAATTTAAGGTTTCTTCCCCCCAACACCTATCACCTAATTTATTTATTATATGTGGTATAAACATTATTTTCCAGCCATTGTTTTCCTATTAATTTTTACCATTTATGGTGGTGATTTTTTGATATTTTACAGTTTTACATGTTAGATTTCCATATCTGACCATTTTAAGTGTTGTGATTTTTTTCTTTCCTTTGATTAGTGTATTCACCTGTATTTTTTTCTAGTTATTTAATTGCTTCATTTTTTACAATTAACTCCTTAATCCACCTAGAATTCATTTTGATATATGGTATACAGTAGGATTTAATTTTTTTTCCCCCTAATAGGTCACCGATTGTTTCAGCATCACTTATTGAATAATCCAACGCAACAACTGTTCTTCCATTAGATAGGGTGTAGCTTTCCACAGGTCGGCCATTTTCTCTTTACCCAAGATGGTTACATTTTGCAATGGTACTCCTACCTTCCTGCAGCTTCTGACAGCATACTAGAGCACCATGTTTGTGCTTGTGCGTTTCAAAAATCCTAGGCACATGCAGATTAGGGCAGTATTTTAAACAGGATTTTTTTTTTCATTTTAAATATGTTAAGTTTGTCACATTCTTGTTGCAAATTAAAATTAAGGAATCCAAATATATGAAACCACGGTTTCAGGTG

Probe1

GGAAGAACAGTTGTTGCGTTGGA

Probe2

TACTGCCCTAATCTGCATGTGCC

Probe3 CAGGTGAATACACTAATCAAAGGAAAGA

GTCCACTTATG

**Smart Silencer target sequences**

AGTGCGACAGACAGCCTCAGAGCTGGTGGGGAGACAGCATGCCACTGGAAAAATTCGTAGACATGGAATTCCTGGATCAAATGGTATGAAACAATTTAAGGTTTCTTCCCCCCAACACCTATCACCTAATTTATTTATTATATGTGGTATAAACATTATTTTCCAGCCATTGTTTTCCTATTAATTTTTACCATTTATGGTGGTGATTTTTTGATATTTTACAGTTTTACATGTTAGATTTCCATATCTGACCATTTTAAGTGTTGTGATTTTTTTCTTTCCTTTGATTAGTGTATTCACCTGTATTTTTTTCTAGTTATTTAATTGCTTCATTTTTTACAATTAACTCCTTAATCCACCTAGAATTCATTTTGATATATGGTATACAGTAGGATTTAATTTTTTTTCCCCCTAATAGGTCACCGATTGTTTCAGCATCACTTATTGAATAATCCAACGCAACAACTGTTCTTCCATTAGATAGGGTGTAGCTTTCCACAGGTCGGCCATTTTCTCTTTACCCAAGATGGTTACATTTTGCAATGGTACTCCTACCTTCCTGCAGCTTCTGACAGCATACTAGAGCACCATGTTTGTGCTTGTGCGTTTCAAAAATCCTAGGCACATGCAGATTAGGGCAGTATTTTAAACAGGATTTTTTTTTTCATTTTAAATATGTTAAGTTTGTCACATTCTTGTTGCAAATTAAAATTAAGGAATCCAAATATATGAAACCACGGTTTCAGGTG

***Smart Silencer target sequences**

**Table 5 Primers list**

| Gene Name | Primers (5'-3') | |
| --- | --- | --- |
| lncRNA MDHDH | TGTAGCTTTCCACAGGTCGG | (Forward) |
|  | ACGCACAAGCACAAACATGG | (Reverse) |
| MDH2 | AGCCGCCTGACCCTCTAT | (Forward) |
|  | AAGGTGTTGGCTCTGACGAT | (Reverse) |
| PSMA1 | GAGGGTATTCCACCCTGCAAA | (Forward) |
|  | AGACCAACTGTGGCTGAACC | (Reverse) |
| ACTB(Beta-actin) | CTTCGCGGGCGACGAT | (Forward) |
|  | CCACATAGGAATCCTTCTGACC | (Reverse) |
| NR_028345(TSS primers for ChIP-seq) | AACAGGGTTCTACCGACAGC | (Forward) |
|  | TCTCCCGTCTCCTAGCAACA | (Reverse) |
| NR_028344.1 | ACTCTCCCAGAGGCACGTAT | (Forward) |
|  | GTGGCCTATGGTGCTTGTCT | (Reverse) |
| NR_173144.1 | CCTGAGTTCCACGATCGCTT | (Forward) |
|  | CTCTCAAAGGCGCGGACTTA | (Reverse) |
| NR_173140.1 | CCTGAGTTCCACGATCGCTT | (Forward) |
|  | CTCTCAAAGGCGCGGACTTA | (Reverse) |
| NR_173139.1 | AATGGGAAGAGGGCTTCGTG | (Forward) |
|  | AAGCGATCGTGGAACTCAGG | (Reverse) |
| NR_104228.1 | CCTGAGTTCCACGATCGCTT | (Forward) |
|  | CTCTCAAAGGCGCGGACTTA | (Reverse) |

**Table 6 ROC baseline data**

| Group | GTEx-Normal | TCGA-GBM | TCGA-LGG |
| --- | --- | --- | --- |
| n | 1157 | 166 | 523 |
| TPM(min) | 0 | 0 | 0 |
| TPM(max) | 7.873 | 4.385 | 4.587 |
| Median | 3.776 | 1.05 | 2.548 |
| IQR | 1.599 | 1.672 | 1.109 |
| Mean | 3.825 | 1.384 | 2.464 |
| SD | 1.292 | 1.093 | 0.86 |
| SE | 0.038 | 0.085 | 0.038 |
| AUC |  | 0.919 | 0.812 |
| CI |  | 0.897-0.941 | 0.792-0.832 |

**Table 7 LINC00632 expression in human glioma patients (TCGA-GBM and LGG) with different clinicopathological features. P values were determined by Chi-square and Wilcoxon tests.**

| Characteristic | Low expression of LINC00632 | High expression of LINC00632 | p |
| --- | --- | --- | --- |
| n | 348 | 348 |  |
| WHO grade, n (%) |  |  | < 0.001 |
| G2 | 87 (13.7%) | 137 (21.6%) |  |
| G3 | 112 (17.6%) | 131 (20.6%) |  |
| G4 | 134 (21.1%) | 34 (5.4%) |  |
| IDH status, n (%) |  |  | < 0.001 |
| WT | 203 (29.6%) | 43 (6.3%) |  |
| Mut | 138 (20.1%) | 302 (44%) |  |
| 1p/19q codeletion, n (%) |  |  | < 0.001 |
| codel | 43 (6.2%) | 128 (18.6%) |  |
| non-codel | 301 (43.7%) | 217 (31.5%) |  |
| Primary therapy outcome, n (%) |  |  | 0.002 |
| PD | 62 (13.4%) | 50 (10.8%) |  |
| SD | 54 (11.7%) | 93 (20.1%) |  |
| PR | 19 (4.1%) | 45 (9.7%) |  |
| CR | 52 (11.3%) | 87 (18.8%) |  |
| Gender, n (%) |  |  | 0.818 |
| Female | 151 (21.7%) | 147 (21.1%) |  |
| Male | 197 (28.3%) | 201 (28.9%) |  |
| Age, n (%) |  |  | < 0.001 |
| <=60 | 243 (34.9%) | 310 (44.5%) |  |
| >60 | 105 (15.1%) | 38 (5.5%) |  |
| Histological type, n (%) |  |  | < 0.001 |
| Astrocytoma | 108 (15.5%) | 87 (12.5%) |  |
| Glioblastoma | 134 (19.3%) | 34 (4.9%) |  |
| Oligoastrocytoma | 44 (6.3%) | 90 (12.9%) |  |
| Oligodendroglioma | 62 (8.9%) | 137 (19.7%) |  |
| OS event, n (%) |  |  | < 0.001 |
| Alive | 161 (23.1%) | 263 (37.8%) |  |
| Dead | 187 (26.9%) | 85 (12.2%) |  |
| DSS event, n (%) |  |  | < 0.001 |
| Alive | 164 (24.3%) | 267 (39.6%) |  |
| Dead | 170 (25.2%) | 74 (11%) |  |
| PFI event, n (%) |  |  | < 0.001 |
| Alive | 130 (18.7%) | 220 (31.6%) |  |
| Dead | 218 (31.3%) | 128 (18.4%) |  |
| Age, meidan (IQR) | 52.5 (38, 63) | 40 (32, 51) | < 0.001 |

**Table 8 Correlation of LINC00632 expression in human glioma patients with different clinicopathological features. Odds Ratio and P value were determined by univariate logistic regression of LINC00632 expression.**

| Characteristics | Total(N) | Odds Ratio(OR) | P value |
| --- | --- | --- | --- |
| WHO grade (G2&G3 vs. G4) | 635 | 4.852 (3.246-7.408) | <0.001 |
| 1p/19q codeletion (non-codel vs. codel) | 689 | 0.242 (0.163-0.354) | <0.001 |
| Primary therapy outcome (PD&SD vs. PR&CR) | 462 | 0.596 (0.407-0.870) | 0.008 |
| IDH status (WT vs. Mut) | 686 | 0.108 (0.074-0.157) | <0.001 |
| Gender (Female vs. Male) | 696 | 0.954 (0.706-1.288) | 0.759 |
| Age (>60 vs. <=60) | 696 | 0.260 (0.170-0.389) | <0.001 |
| Histological type (Astrocytoma&Glioblastoma vs. Oligoastrocytoma&Oligodendroglioma) | 696 | 0.216 (0.156-0.296) | <0.001 |

**Table 9 Univariate and multivariate Cox regression of LINC00632 expression for overall survival (OS) in glioma patients.**

| Characteristics | Total(N) | Univariate analysis | |  | Multivariate analysis | |
| --- | --- | --- | --- | --- | --- | --- |
|  |  | Hazard ratio (95% CI) | P value |  | Hazard ratio (95% CI) | P value |
| Gender | 695 |  |  |  |  |  |
| Female | 297 | Reference |  |  |  |  |
| Male | 398 | 1.262 (0.988-1.610) | 0.062 |  | 1.119 (0.864-1.448) | 0.394 |
| Age | 695 |  |  |  |  |  |
| <=60 | 552 | Reference |  |  |  |  |
| >60 | 143 | 4.668 (3.598-6.056) | **<0.001** |  | 2.952 (2.235-3.900) | **<0.001** |
| Histological type | 695 |  |  |  |  |  |
| Astrocytoma&Glioblastoma | 363 | Reference |  |  |  |  |
| Oligoastrocytoma&Oligodendroglioma | 332 | 0.267 (0.204-0.350) | **<0.001** |  | 0.439 (0.320-0.601) | **<0.001** |
| WHO grade | 634 |  |  |  |  |  |
| G2 | 223 | Reference |  |  |  |  |
| G3&G4 | 411 | 5.642 (3.926-8.109) | **<0.001** |  | 3.427 (2.331-5.038) | **<0.001** |
| LINC00632 | 695 |  |  |  |  |  |
| Low | 348 | Reference |  |  |  |  |
| High | 347 | 0.406 (0.315-0.524) | **<0.001** |  | 0.713 (0.531-0.958) | **0.025** |

**Table 10 Univariate and multivariate Cox regression of LINC00632 expression for disease specific survival (DSS) in glioma patients.**

| Characteristics | Total(N) | Univariate analysis | |  | Multivariate analysis | |
| --- | --- | --- | --- | --- | --- | --- |
|  |  | Hazard ratio (95% CI) | P value |  | Hazard ratio (95% CI) | P value |
| Gender | 674 |  |  |  |  |  |
| Female | 289 | Reference |  |  |  |  |
| Male | 385 | 1.248 (0.965-1.614) | 0.092 |  | 1.093 (0.831-1.438) | 0.524 |
| Age | 674 |  |  |  |  |  |
| <=60 | 541 | Reference |  |  |  |  |
| >60 | 133 | 4.500 (3.409-5.940) | **<0.001** |  | 2.788 (2.071-3.754) | **<0.001** |
| Histological type | 674 |  |  |  |  |  |
| Astrocytoma&Glioblastoma | 347 | Reference |  |  |  |  |
| Oligoastrocytoma&Oligodendroglioma | 327 | 0.256 (0.192-0.340) | **<0.001** |  | 0.423 (0.303-0.591) | **<0.001** |
| WHO grade | 614 |  |  |  |  |  |
| G2 | 220 | Reference |  |  |  |  |
| G3&G4 | 394 | 5.816 (3.956-8.551) | **<0.001** |  | 3.534 (2.346-5.324) | **<0.001** |
| LINC00632 | 674 |  |  |  |  |  |
| Low | 337 | Reference |  |  |  |  |
| High | 337 | 0.396 (0.302-0.520) | **<0.001** |  | 0.677 (0.493-0.928) | **0.015** |

**Table 11 Univariate and multivariate Cox regression of LINC00632 expression for progress free interval (PFI) in glioma patients.**

| Characteristics | Total(N) | Univariate analysis | |  | Multivariate analysis | |
| --- | --- | --- | --- | --- | --- | --- |
|  |  | Hazard ratio (95% CI) | P value |  | Hazard ratio (95% CI) | P value |
| Gender | 695 |  |  |  |  |  |
| Female | 297 | Reference |  |  |  |  |
| Male | 398 | 1.083 (0.875-1.342) | 0.463 |  |  |  |
| Age | 695 |  |  |  |  |  |
| <=60 | 552 | Reference |  |  |  |  |
| >60 | 143 | 2.873 (2.268-3.640) | **<0.001** |  | 2.008 (1.556-2.591) | **<0.001** |
| Histological type | 695 |  |  |  |  |  |
| Astrocytoma&Glioblastoma | 363 | Reference |  |  |  |  |
| Oligoastrocytoma&Oligodendroglioma | 332 | 0.345 (0.275-0.433) | **<0.001** |  | 0.506 (0.386-0.663) | **<0.001** |
| WHO grade | 634 |  |  |  |  |  |
| G2 | 223 | Reference |  |  |  |  |
| G3&G4 | 411 | 2.751 (2.112-3.583) | **<0.001** |  | 1.890 (1.417-2.521) | **<0.001** |
| LINC00632 | 695 |  |  |  |  |  |
| Low | 348 | Reference |  |  |  |  |
| High | 347 | 0.449 (0.361-0.560) | **<0.001** |  | 0.647 (0.501-0.836) | **<0.001** |
