# Supplementary figures and images for: A novel lncRNA MDHDH suppresses glioblastoma multiforme by acting as a scaffold for MDH2 and PSMA1 to regulate NAD+ metabolism and autophagy
Source: J Exp Clin Cancer Res. 2022 Dec 17;41:349. doi: 10.1186/s13046-022-02543-7 (PMC9758949; doi:10.1186/s13046-022-02543-7)

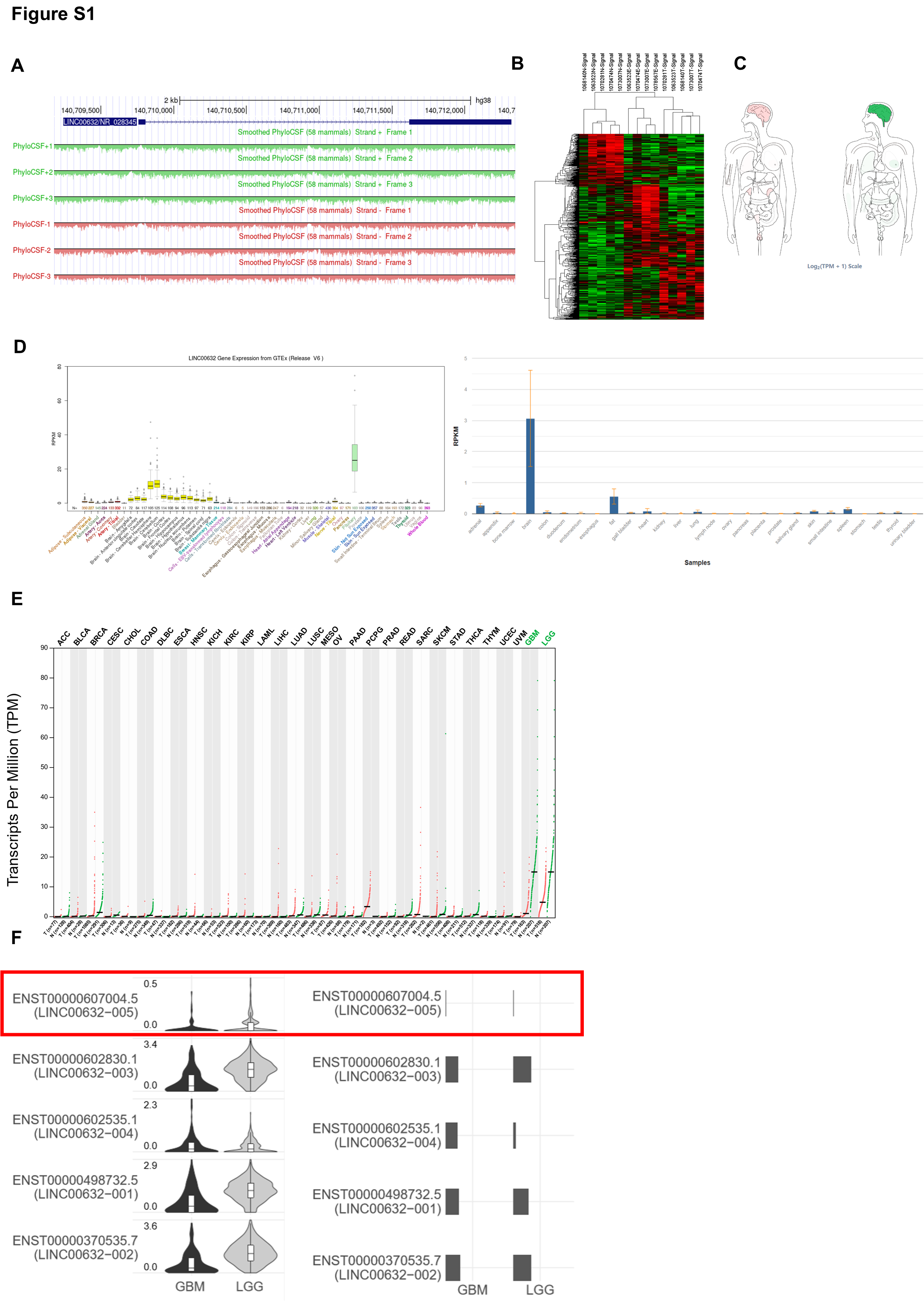

Supplement: Supplementary file 8 — Additional file 8. [file 13046_2022_2543_MOESM8_ESM.tif]

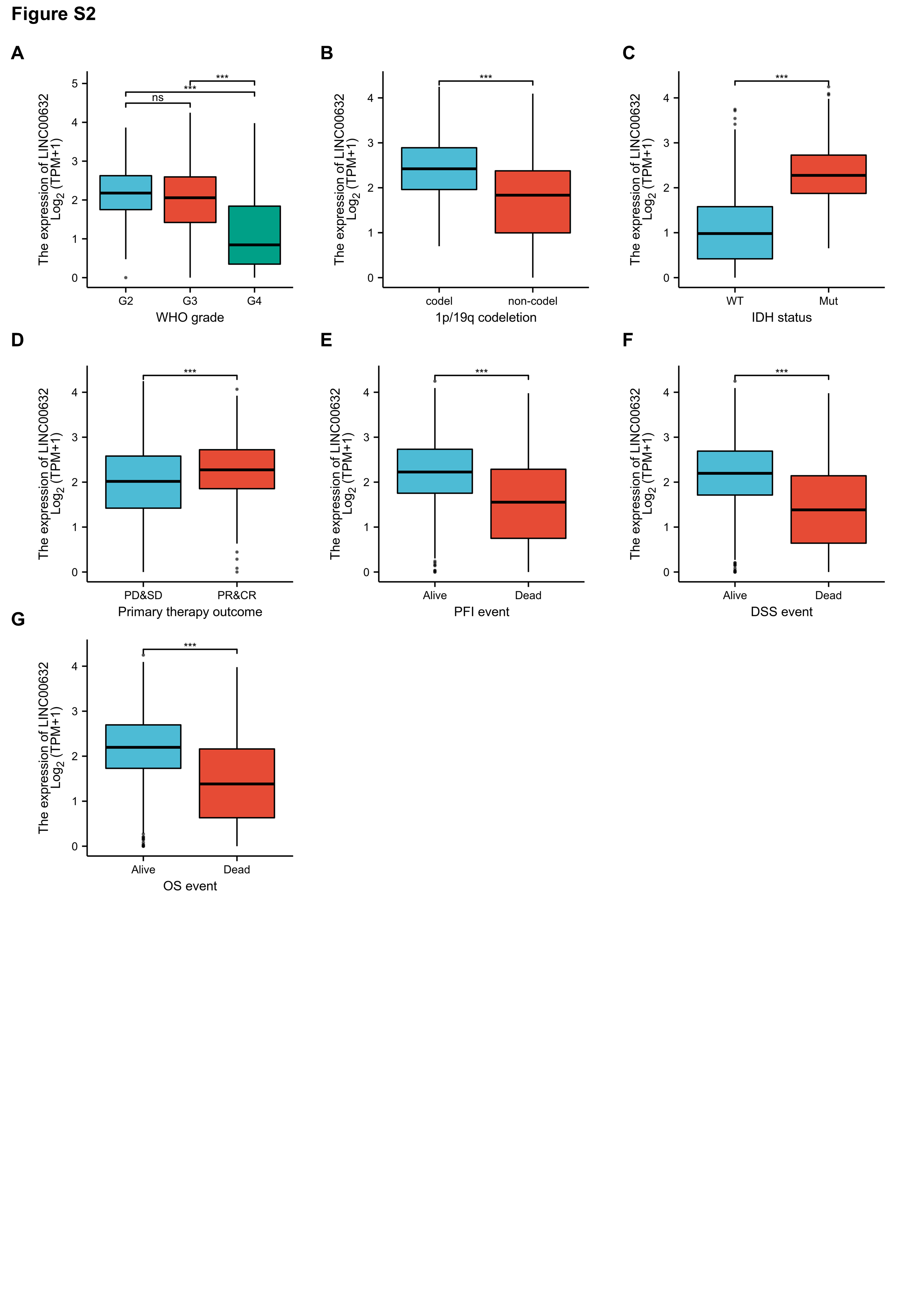

Supplement: Supplementary file 9 — Additional file 9. [file 13046_2022_2543_MOESM9_ESM.tif]

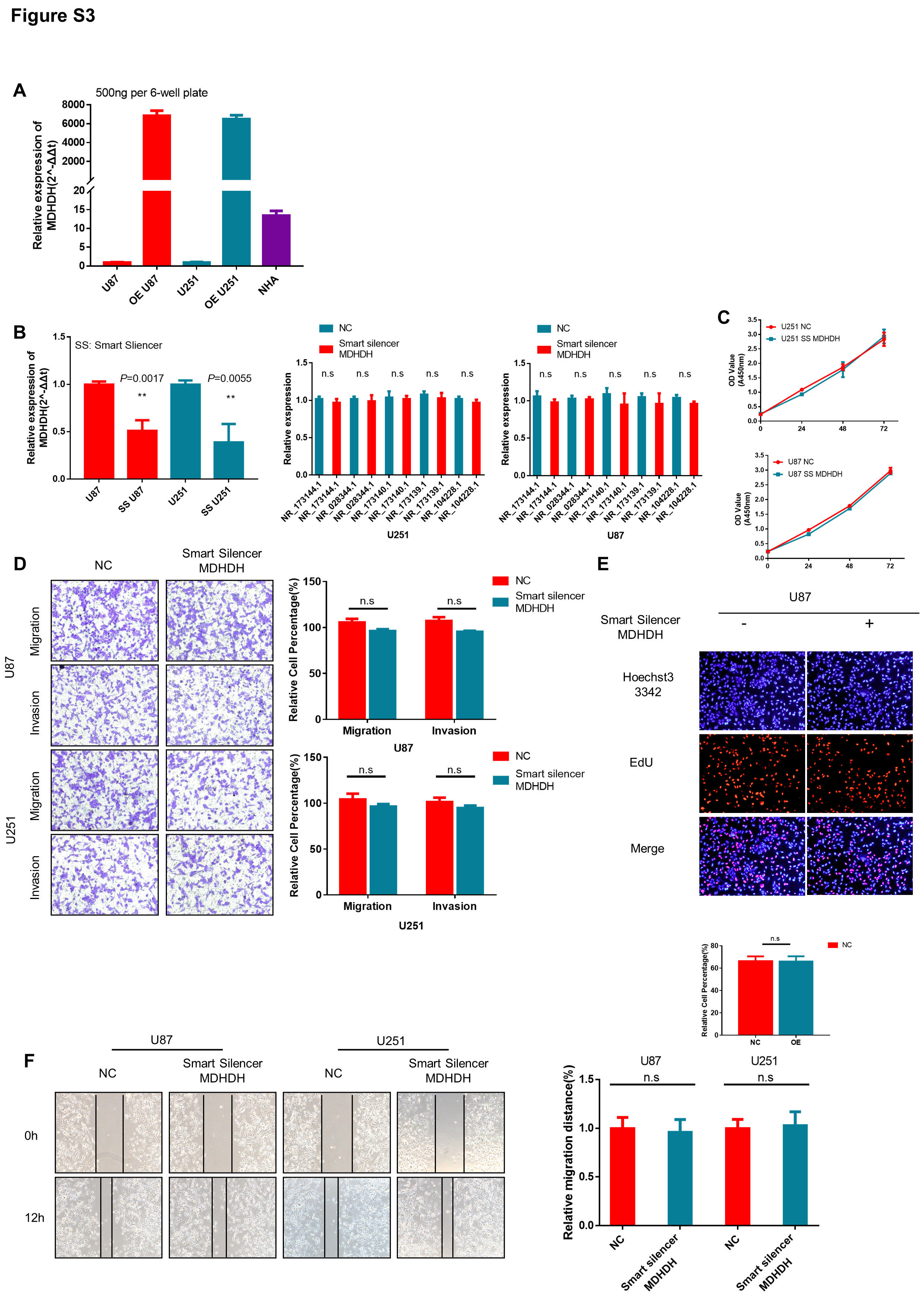

Supplement: Supplementary file 10 — Additional file 10. [file 13046_2022_2543_MOESM10_ESM.tif]

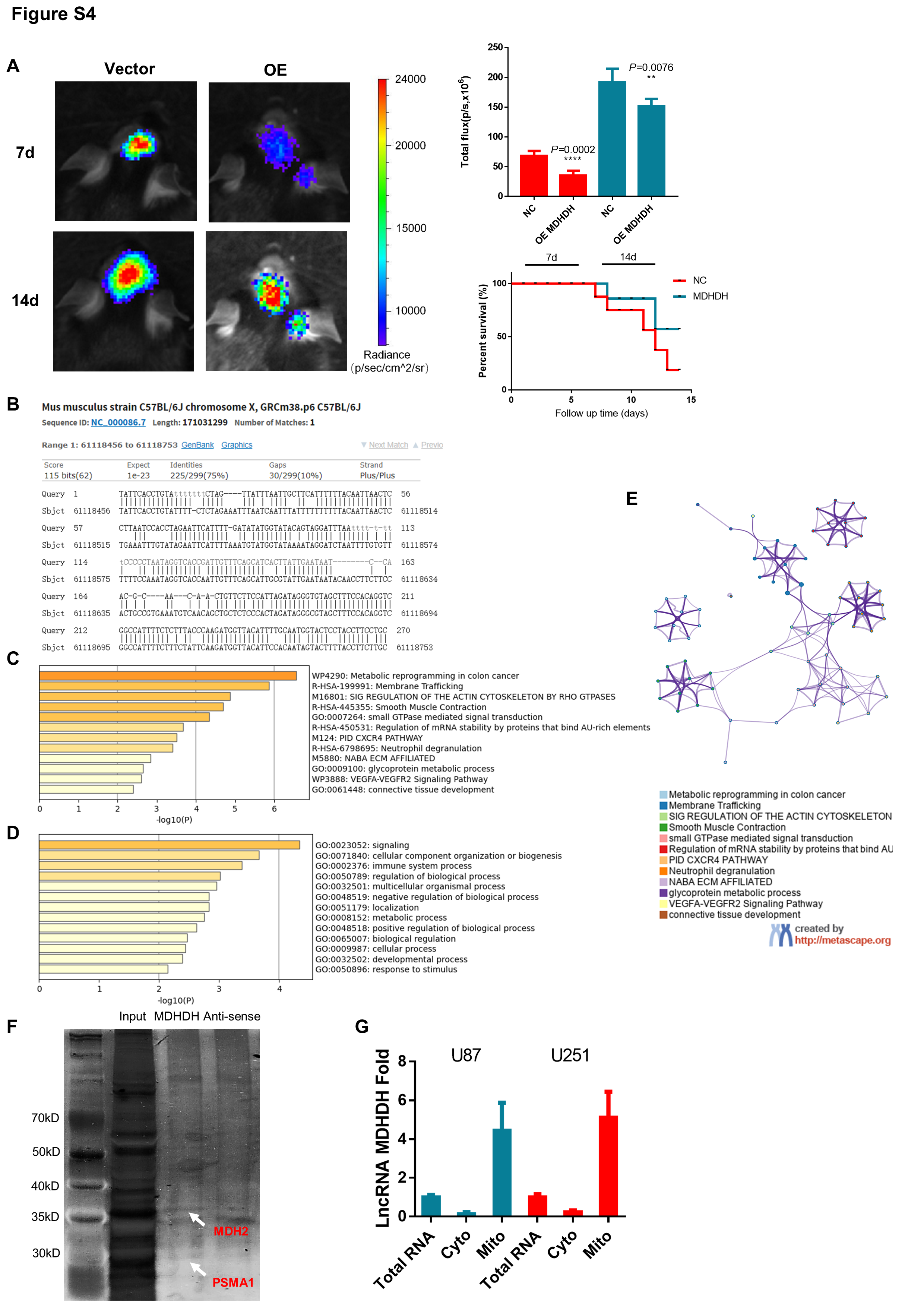

Supplement: Supplementary file 11 — Additional file 11. [file 13046_2022_2543_MOESM11_ESM.tif]

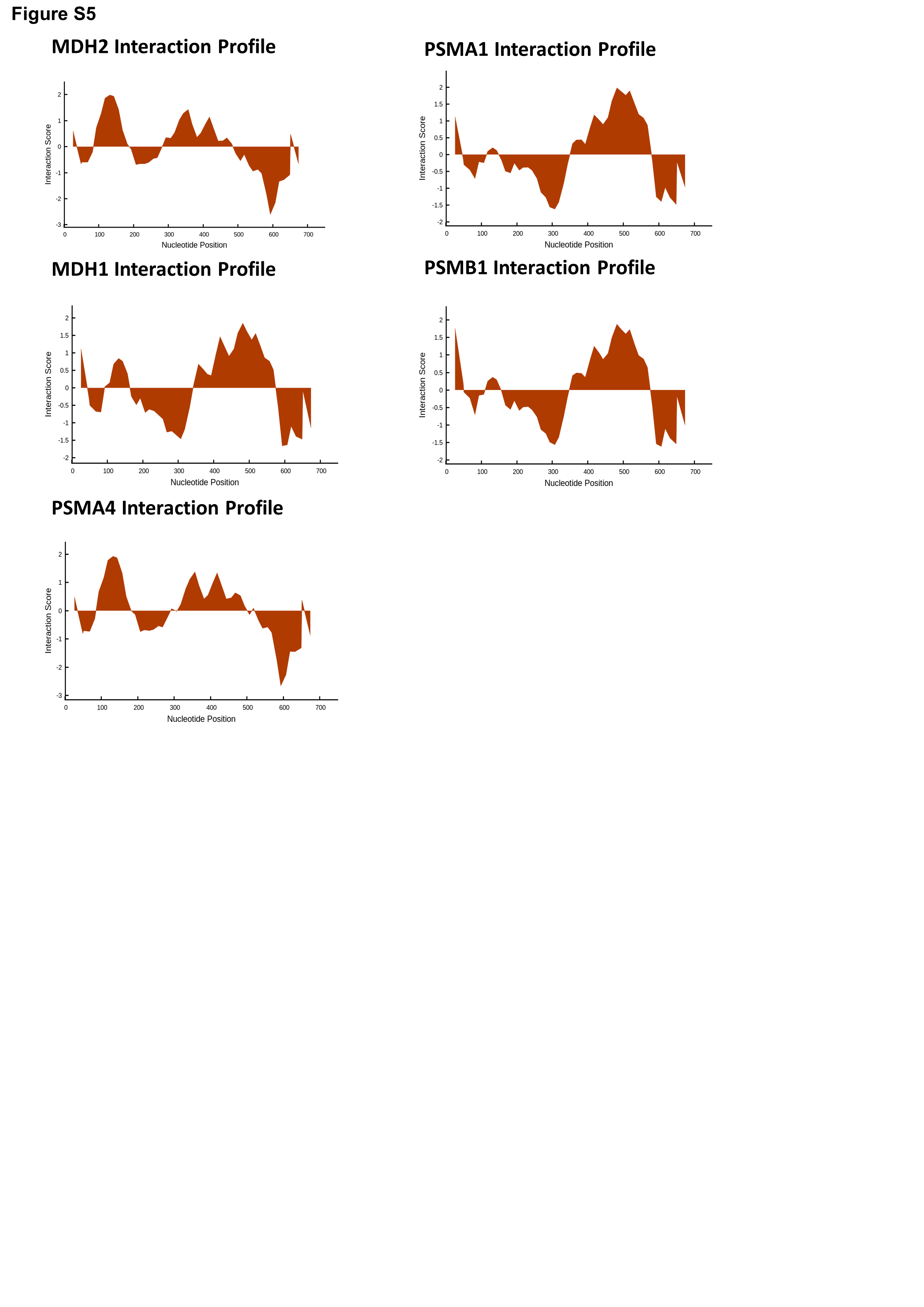

Supplement: Supplementary file 12 — Additional file 12. [file 13046_2022_2543_MOESM12_ESM.tif]

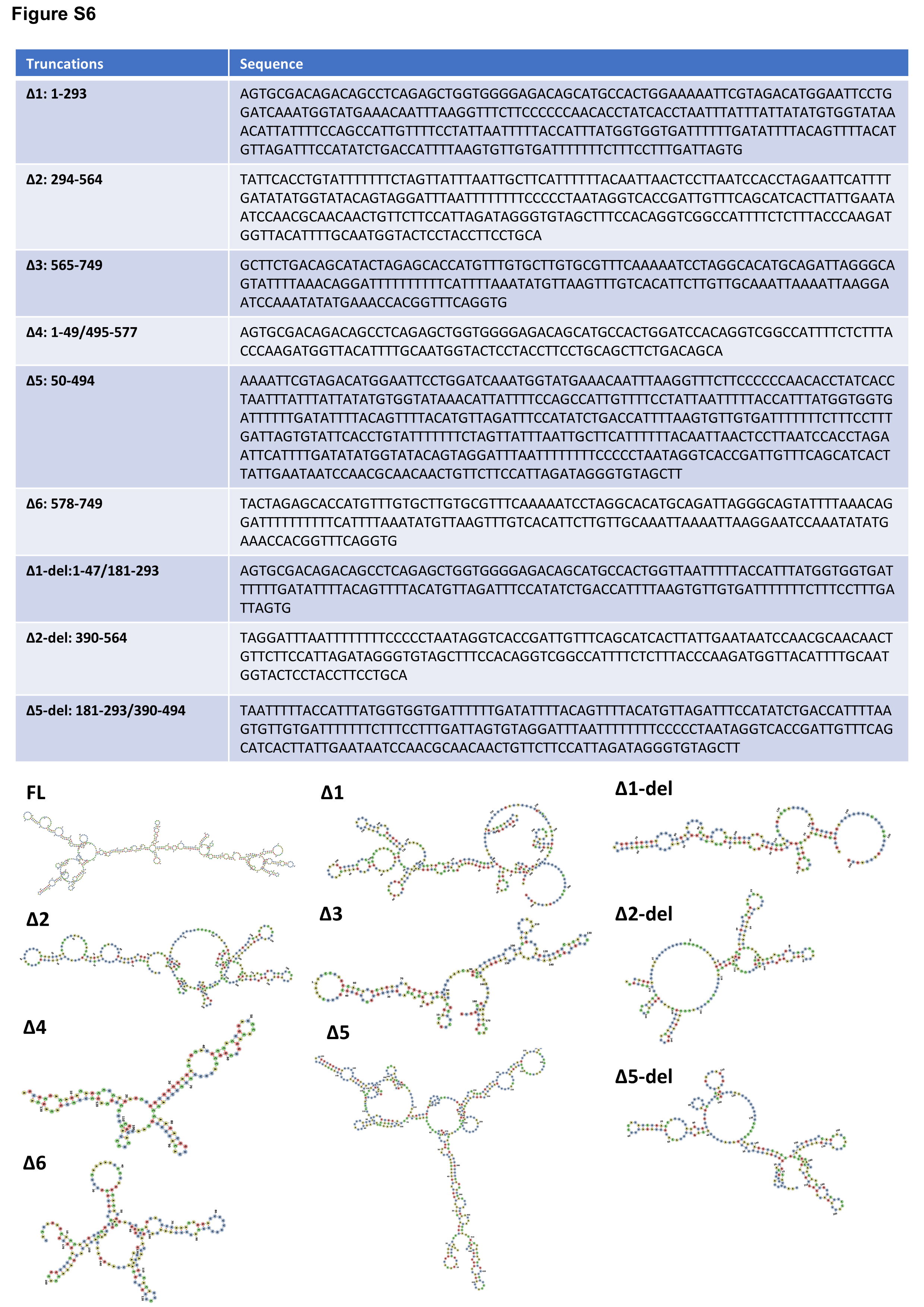

Supplement: Supplementary file 13 — Additional file 13. [file 13046_2022_2543_MOESM13_ESM.tif]

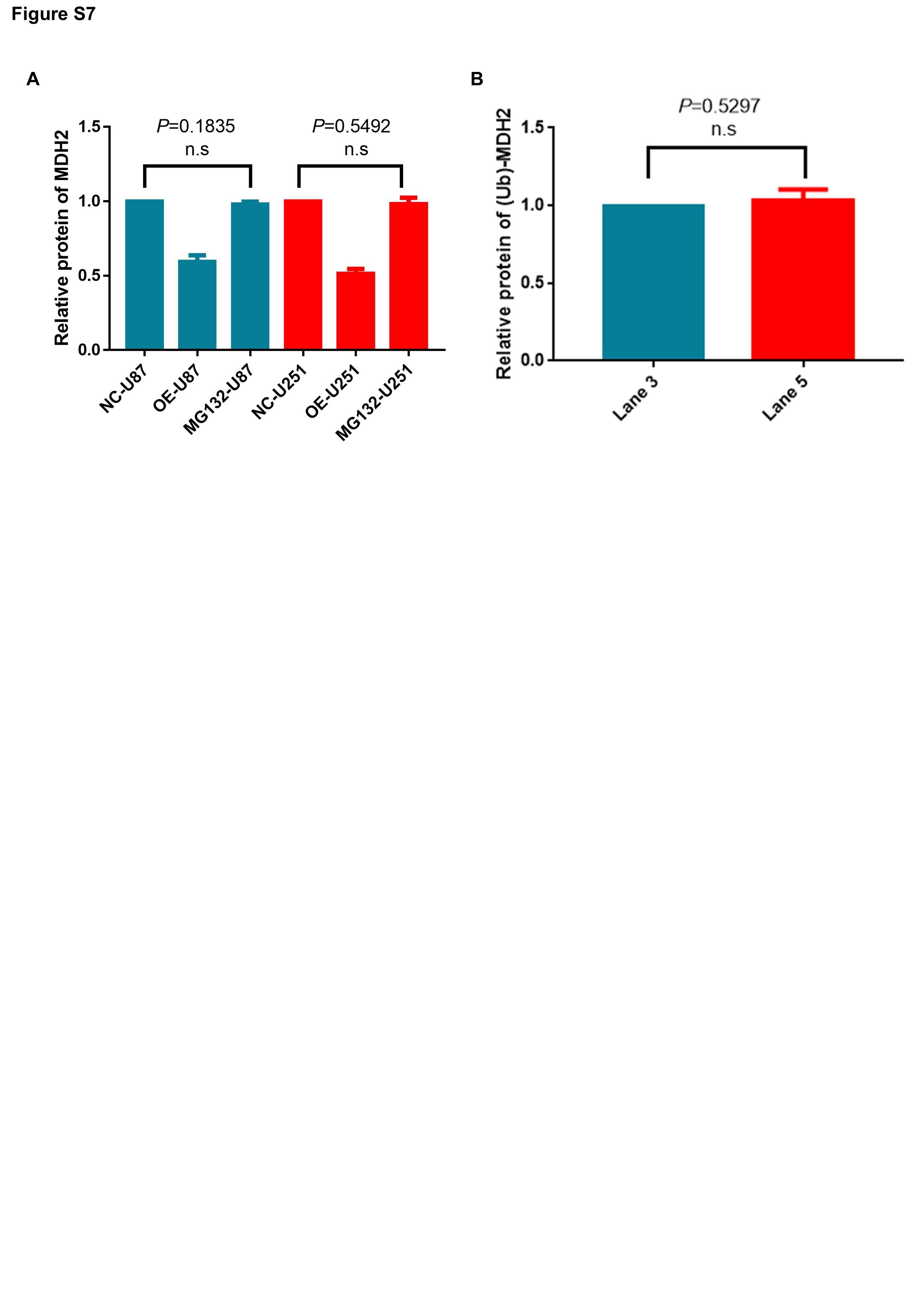

Supplement: Supplementary file 14 — Additional file 14. [file 13046_2022_2543_MOESM14_ESM.tif]

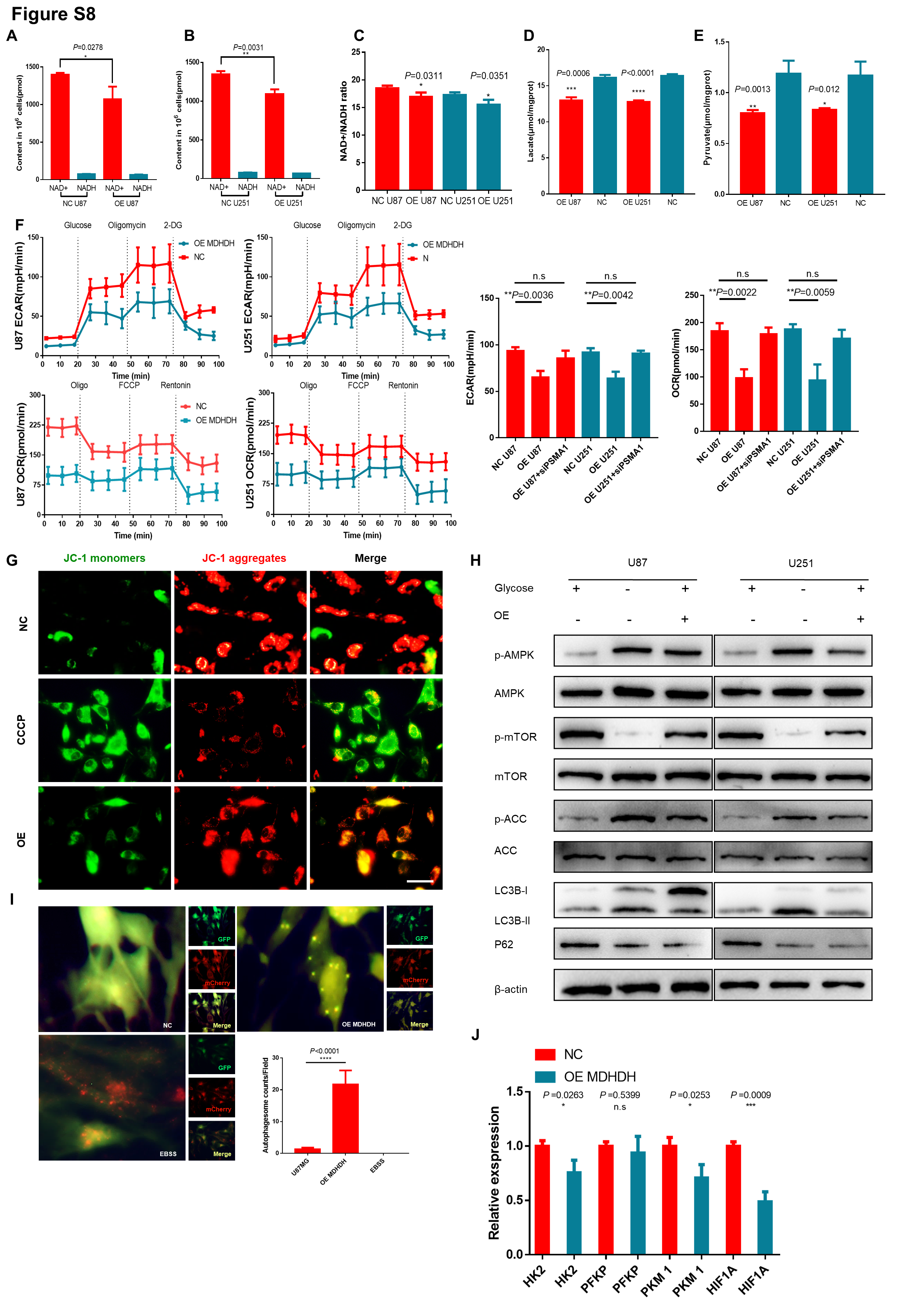

Supplement: Supplementary file 15 — Additional file 15. [file 13046_2022_2543_MOESM15_ESM.tif]

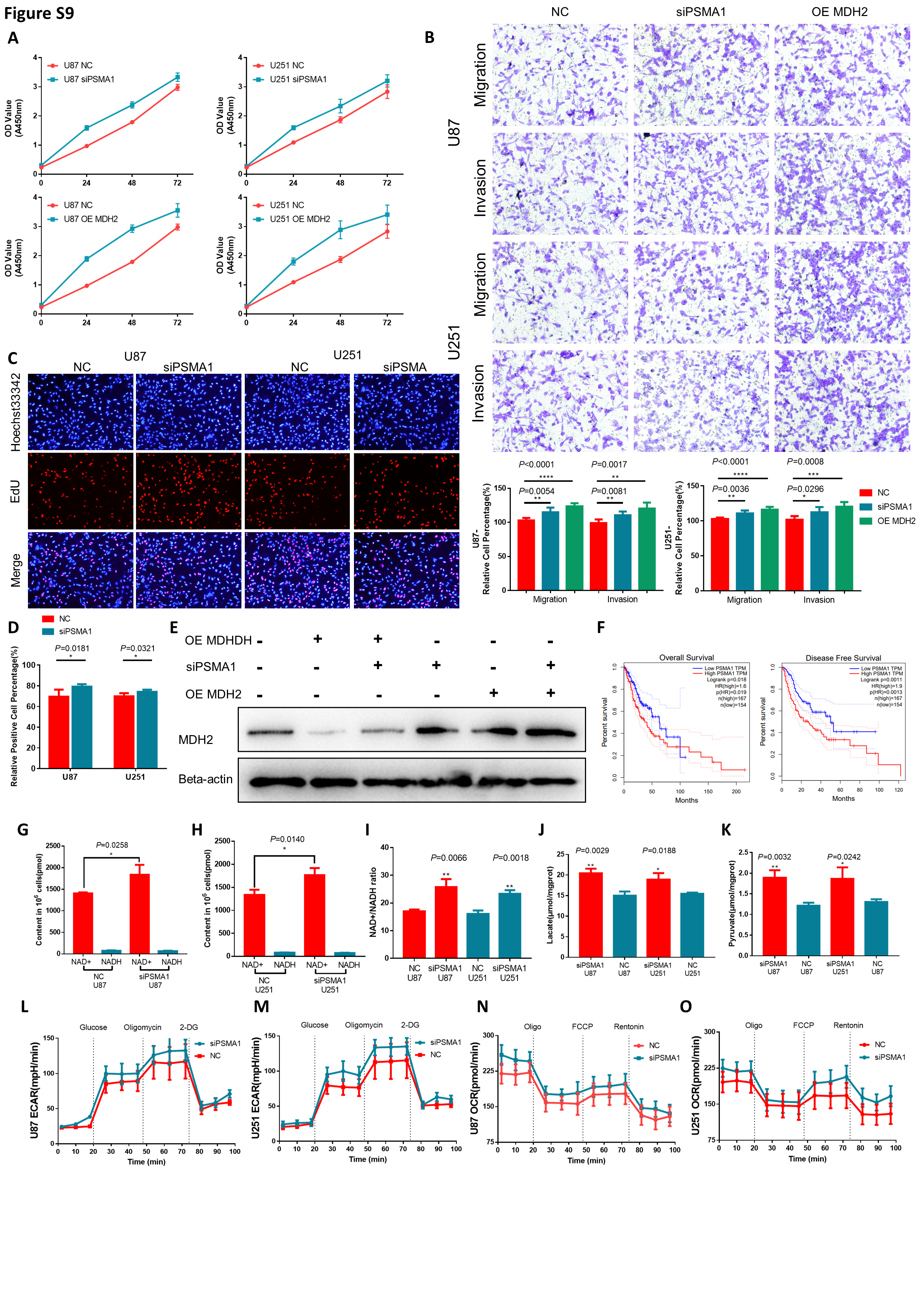

Supplement: Supplementary file 16 — Additional file 16. [file 13046_2022_2543_MOESM16_ESM.tif]

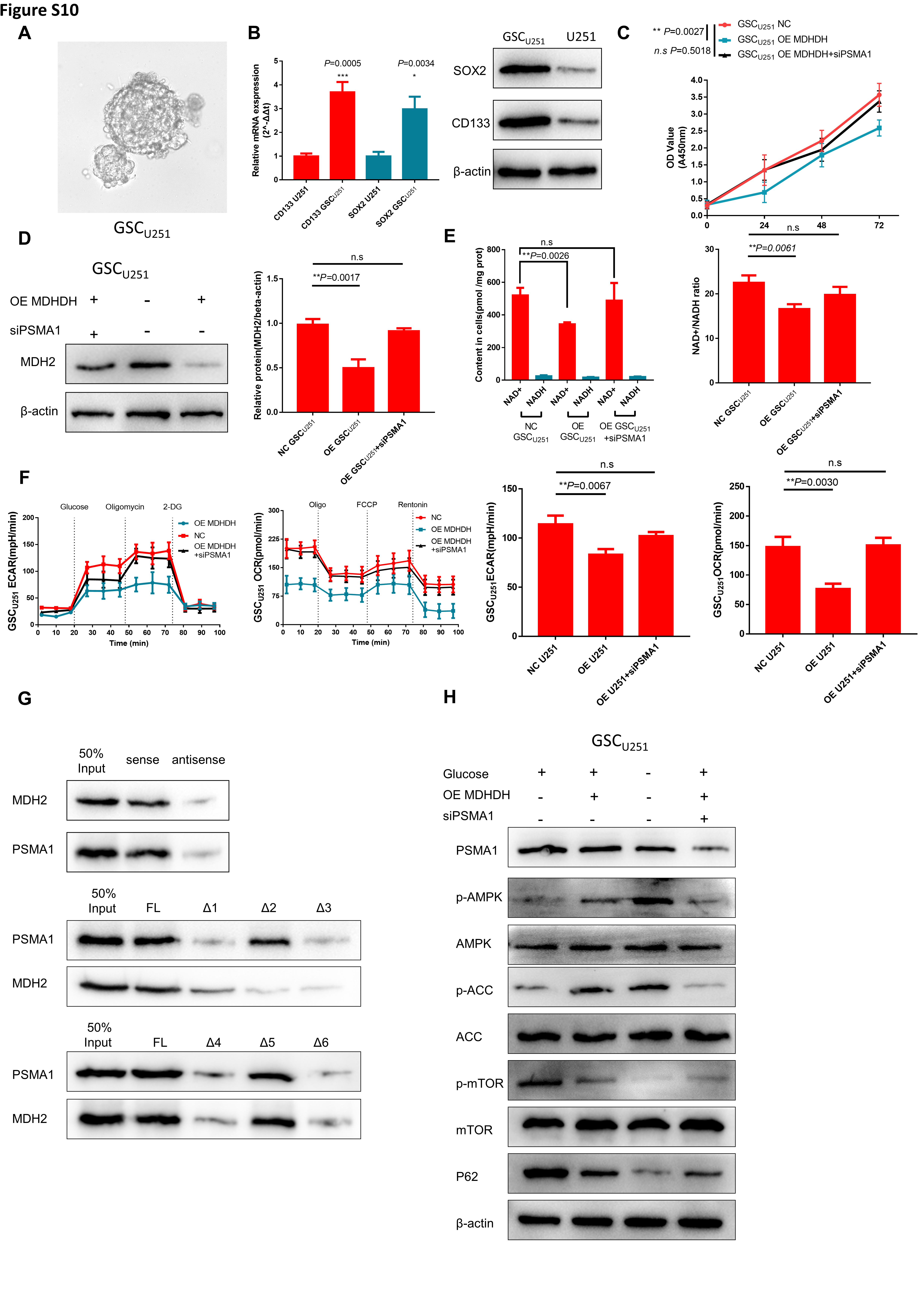

Supplement: Supplementary file 17 — Additional file 17. [file 13046_2022_2543_MOESM17_ESM.tif]

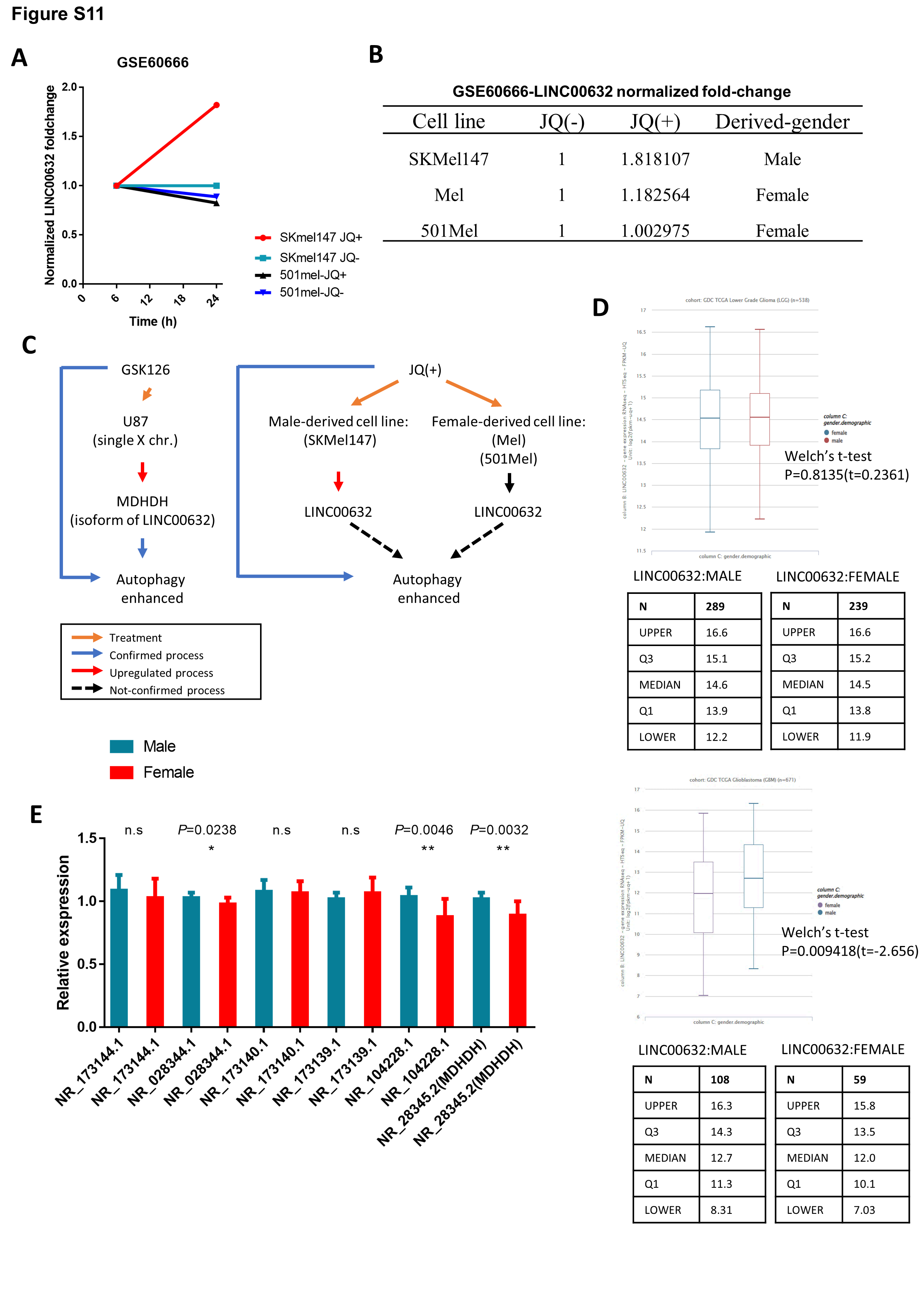

Supplement: Supplementary file 18 — Additional file 18. [file 13046_2022_2543_MOESM18_ESM.tif]

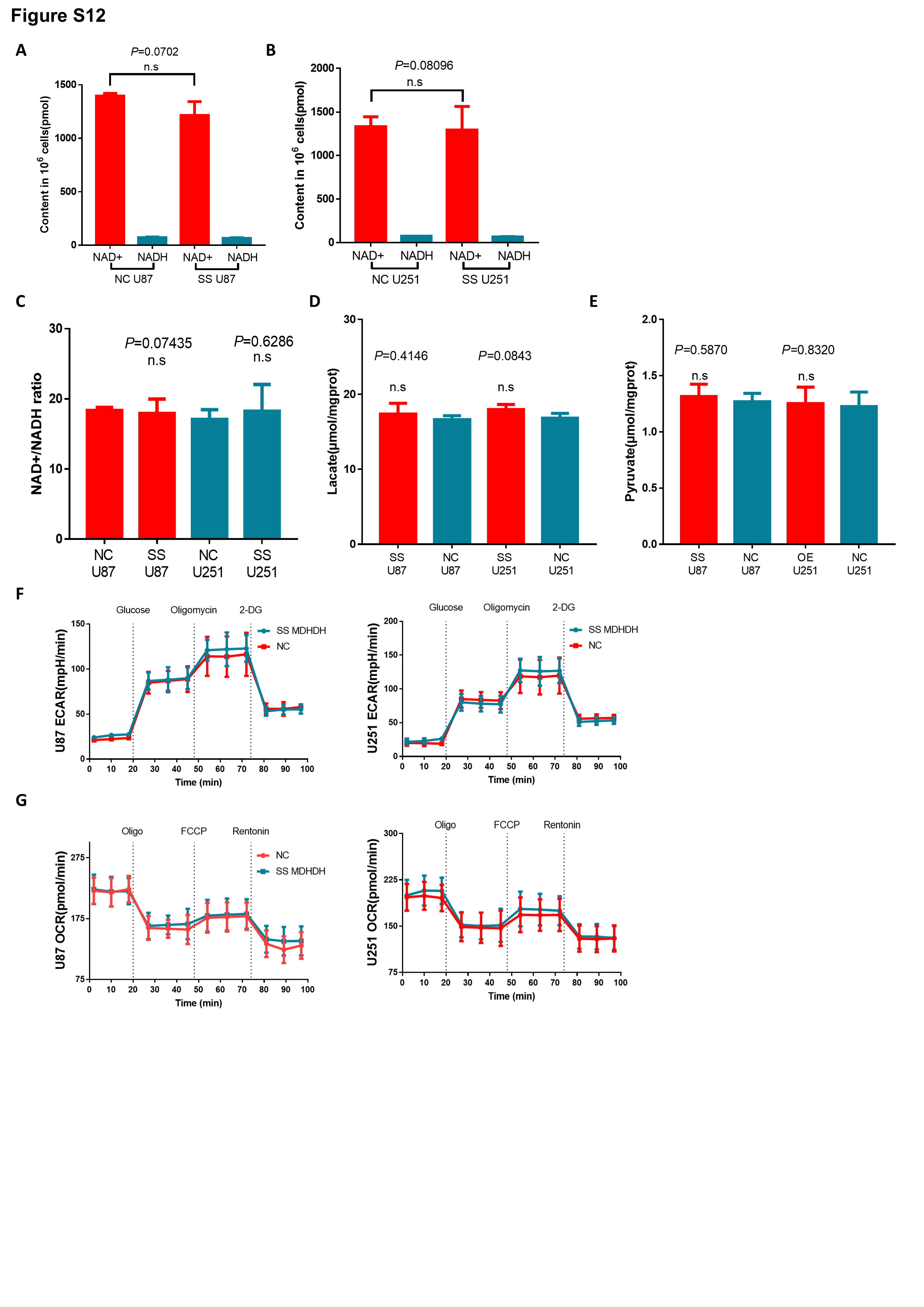

Supplement: Supplementary file 19 — Additional file 19. [file 13046_2022_2543_MOESM19_ESM.tif]

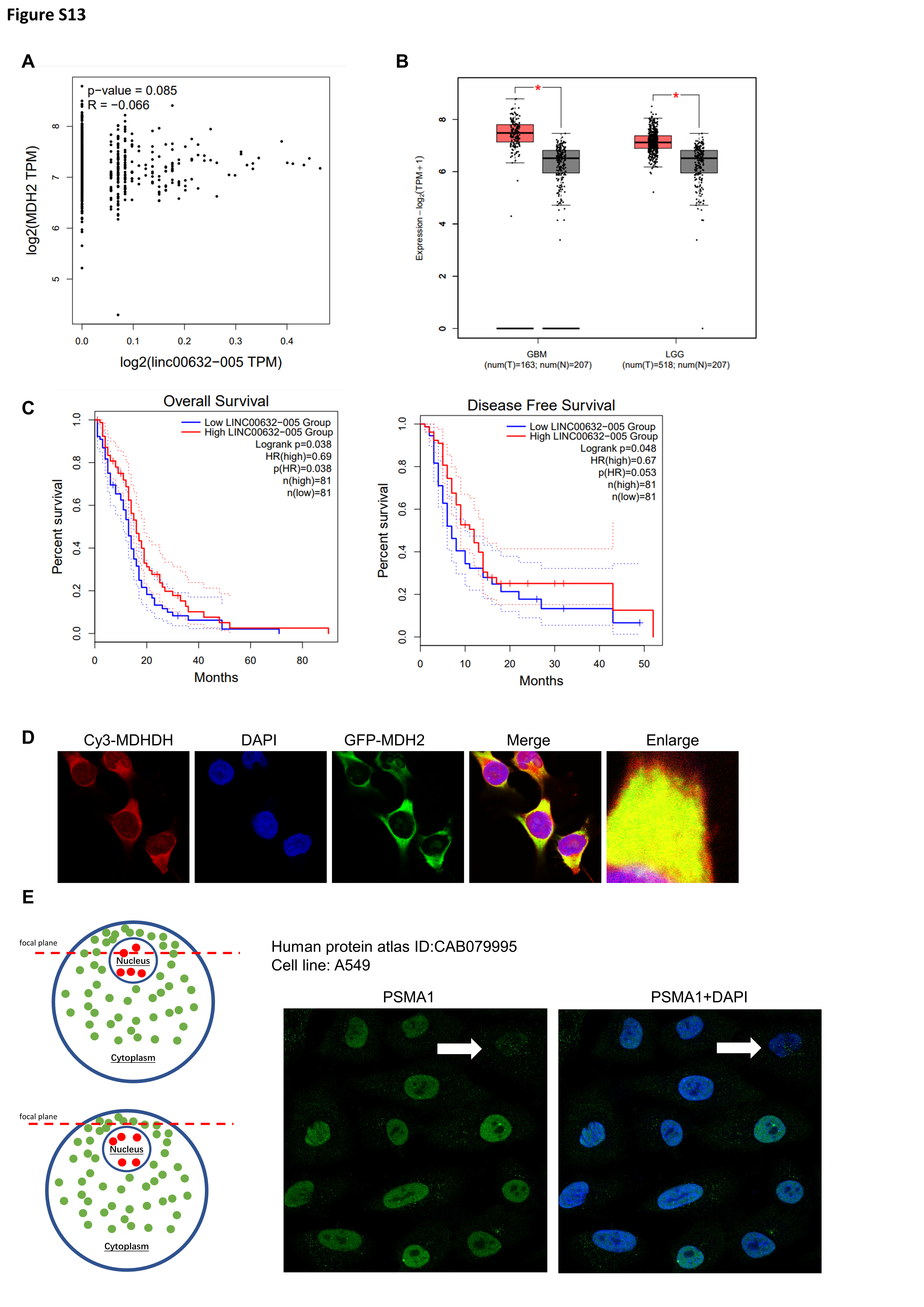

Supplement: Supplementary file 20 — Additional file 20. [file 13046_2022_2543_MOESM20_ESM.tif]
